# Molecular Regulators of In Vitro Regeneration in Wheat: Roles of Morphogenic Factors in Transformation, Genome Editing, and Breeding

**DOI:** 10.3390/ijms27031271

**Published:** 2026-01-27

**Authors:** Sylwia Kowalik, Monika Samoń, Mateusz Przyborowski

**Affiliations:** Plant Breeding and Acclimatization Institute - National Research Institute, Radzików, 05-870 Błonie, Poland; m.samon@ihar.edu.pl

**Keywords:** *Triticum aestivum*, in vitro regeneration, somatic embryogenesis, morphogenic regulators, BBM, WUS2, GRF-GIF, CLE/CLV-WUS signaling, CRISPR-Cas genome editing

## Abstract

Efficient in vitro regeneration remains a major constraint in the genetic transformation, genome editing, and molecular breeding of wheat (*Triticum aestivum* L.), largely due to strong genotype-dependent recalcitrance and limited activation of developmental programs required for somatic embryogenesis. Plant regeneration relies on extensive transcriptional reprogramming and epigenetic remodeling orchestrated by morphogenic regulators that modulate meristem identity, as well as cellular pluri- and totipotency. In this review, we synthesize current molecular knowledge on key transcription factors (*BBM*, *WUS/WUS2*, *GRF-GIF*, *WOX*, *LAX1*, *SERK*, *WIND1/ERF115*) and signaling peptides (*CLE/CLV-WUS* module, phytosulfokine/*PSK*) that regulate embryogenic competence in monocot cereals, with emphasis on their orthologs and functional relevance in wheat. We highlight how controlled expression of these morphogenic genes, promoter engineering, and transient or excisable induction systems can significantly enhance regeneration capacity, reduce chimerism in CRISPR-Cas-edited plants, and facilitate genotype-independent transformation. We also discuss epigenetic and metabolic constraints underlying wheat recalcitrance and their potential modulation to improve culture responsiveness. By integrating evidence from wheat, rice, maize, and barley, we outline conserved gene-regulatory networks that reinitiate totipotency and propose strategies to accelerate doubled haploid production and speed-breeding pipelines. Collectively, morphogenic factors emerge as central molecular tools for overcoming regeneration bottlenecks and enabling next-generation wheat improvement. The objective of this review is to synthesize and critically evaluate current molecular knowledge on morphogenic regulators controlling in vitro regeneration in wheat (*Triticum aestivum* L.), with particular emphasis on their roles in genetic transformation and genome editing.

## 1. Introduction

### 1.1. The Importance of In Vitro Regeneration for Transformation, Genome Editing, and Wheat Breeding

In vitro regeneration is a fundamental step in plant biotechnology, allowing the recovery of whole plants from individual cells or explant-derived tissues. In wheat, this process represents the biological bottleneck determining whether genome-edited or transformed cells can develop into fertile plants. This capacity underpins both genetic transformation and genome-editing technologies, including CRISPR-Cas9. In *Triticum aestivum*, one of the major global cereal crops, efficient regeneration is a prerequisite for the recovery of stable and fertile plants with modified phenotypes and hence for the implementation of breeding programs targeting enhanced yield, stress resilience, and grain quality [1,2]. Importantly, the efficiency of in vitro regeneration is strongly influenced by environmental factors, such as light, temperature, and culture medium composition, which regulate key developmental processes, including germination, morphogenesis, and plantlet development [3,4].

The ability to regenerate entire plants from somatic tissues relies on the reactivation of molecular pathways that re-establish pluripotency. Plants exhibit remarkable regenerative competence derived from their intrinsic cellular totipotency. At the molecular level, regeneration entails extensive transcriptional reprogramming and epigenetic remodeling, which collectively re-establish cellular pluripotency. De novo regeneration involves, among other things, the following mechanisms: transcription factors *LBD* (*LATERAL ORGAN BOUNDARIES DOMAIN*), *WOX* (*WUSCHEL*—RELATED HOMEBOX), *PLT* (*PLETHORA*), *ARR* (*ARABIDOPSIS RESPONSE REGULATORS*), and *BBM* (*BABY BOOM*), which modulate auxin and cytokinin signaling networks [5,6].

The importance of regeneration for breeding practice is twofold: firstly, it provides a tool for micropropagation of elite genotypes, and secondly, it serves as an intermediate step in genetic engineering processes. Without an effective regeneration step, genetic transformation or genome editing remains incomplete because the modified cells cannot develop into mature, reproductively capable plants [7].

Moreover, in vitro regeneration constitutes a crucial component in the production of doubled haploid (DH) lines, which represent a cornerstone of modern wheat breeding. DH technology allows for the rapid development of completely homozygous lines within a single generation through chromosome-doubling of haploid plants obtained by anther or microspore culture [8]. The success of this approach depends primarily on the efficiency of androgenesis and subsequent plant regeneration, which are significantly influenced by the genotype, the physiological state of the donor plants, and the specific culture and induction conditions used [9,10]. Regenerated DH plants provide genetically uniform material for precise selection and phenotypic evaluation, and their integration with genome editing and accelerated breeding approaches enables the generation of homozygous edited lines within a single generation [11,12].

### 1.2. Constraints and Genotype Dependency of Regeneration

The efficiency of in vitro wheat regeneration is significantly genotype-dependent. Even under optimized culture conditions, different cultivars display varying capacities for callus formation, somatic embryogenesis, and shoot regeneration [13]. Commonly used model genotypes, such as ‘Bobwhite’ and ‘Fielder’, exhibit high regenerative competence, which makes them widely employed models in studies of wheat transformation and gene function analysis using loss-of-function strategies [14,15,16]. Research on ‘Bobwhite’ conducted mainly between 1992 and 2002 established the first stable wheat transformation systems, including biolistic transformation in 1992 and *Agrobacterium tumefaciens*-mediated transformation in 1997, culminating in the identification of highly transformable ‘Bobwhite’-derived lines in 2002 [17,18]. In contrast, ‘Fielder’ has been intensively exploited since 2014–2015, following the development of high-efficiency *Agrobacterium*-based transformation protocols compatible with CRISPR-Cas genome editing, further supported by the availability of its chromosome-scale genome assembly published in 2021 [19,20,21].

These genotypes have been established as model systems due to their consistently high transformability rather than their performance in a defined year of development. However, despite their utility, ‘Bobwhite’ and ‘Fielder’ possess limited agronomic value and do not represent the phenotypic diversity of breeding material. In contrast, elite cultivars with desirable breeding traits often demonstrate low or unstable in vitro regeneration capacity. Consequently, protocols optimized for high-competence model lines often fail when applied to elite cultivars, highlighting the lack of universal regeneration methods for wheat. This genotype-dependent regeneration ability, known as recalcitrance, represents one of the major bottlenecks in wheat biotechnology [22,23]. Genotype-dependent regeneration reflects variation in the expression of morphogenetic genes (such as *WUS* (*WUSCHEL*), *BBM*, and *SERK* (SOMATIC EMBRYOGENESIS RECEPTOR-LIKE KINASE)), differential hormonal responsiveness, and distinct epigenetic states influencing cellular plasticity.

Moreover, the extensive genetic diversity present in wheat germplasm, including landraces and wild relatives, contributes to variation in regeneration responses. For example, differential regeneration frequencies among genotypes and explants have been repeatedly documented, indicating that the donor plant’s genetic background influences in vitro culture outcomes [24,25]. Environmental conditions experienced by donor plants prior to explant isolation, such as growth temperature and vernalization history, have also been shown to significantly affect regeneration capacity in wheat explants, likely through physiological conditioning of tissues and modulation of developmental competence [26,27,28]. These genotype × environment interactions further constrain the establishment of broadly applicable in vitro regeneration protocols and underscore the necessity of tailored culture strategies across diverse wheat genetic resources.

### 1.3. The Concept and Functional Application of Morphogens in Plants

In plant biology, morphogens are signaling molecules or transcription factors that control cell fate by triggering developmental reprogramming. Because recalcitrant wheat genotypes often fail to activate relevant morphogenetic pathways on their own during regeneration, controlled expression of morphogens can temporarily “unlock” cellular competence and enable regeneration. Key morphogens, such as *WUS*, *BBM*, *LEC1*/*LEC2* (*LEAFY COTYLEDON*), and *SERK*, orchestrate somatic embryogenesis and de novo organogenesis by activating conserved developmental gene networks [29]. In biotechnological applications, morphogens are utilized to enhance the efficiency of the regeneration process. Overexpression of *WUS* and *BBM* genes in wheat tissues markedly enhances the induction of embryogenic callus, shortens the regeneration period, and enables the recovery of transgenic plants even in genotypes previously considered recalcitrant [30]. Because constitutive expression of morphogenes can severely disrupt shoot and root development, controlled or transient expression systems are essential for practical use. These include the use of inducible or tissue-specific promoters, as well as site-specific recombination systems such as Cre-loxP, which allow precise insertion or excision of transgene fragments following successful transformation [31,32]. Pleiotropic effects in this context refer to the influence of *WUS* and *BBM* overexpression on multiple developmental pathways and morphological traits beyond somatic embryogenesis, such as altered organogenesis or abnormal growth patterns. Therefore, precise temporal regulation of morphogen expression is essential to enhancing regenerative competence without compromising normal plant architecture and fertility.

## 2. Wheat Regeneration Recalcitrance

### 2.1. Characterization of Regeneration Recalcitrance in Wheat

Wheat regeneration recalcitrance refers to the limited ability of certain genotypes to dedifferentiate and reinitiate developmental programs required for somatic embryogenesis and shoot organogenesis. Compared with model species such as rice (*Oryza sativa*) or tobacco (*Nicotiana tabacum*), wheat exhibits considerably lower in vitro regeneration efficiency, which represents a major bottleneck for genetic engineering and genome-editing approaches, including CRISPR-Cas-mediated technologies for genome editing [33].

The first successful attempts to generate transgenic wheat date back to the early 1990s [34,35]. Although both *Agrobacterium tumefaciens*-mediated and biolistic transformation protocols have been developed, the regeneration efficiency in many cases has remained below 5% of inoculated explants [36]. Consequently, the progress of wheat genetic transformation has lagged significantly behind that of other cereals, such as rice and maize (*Zea mays*), which display much higher morphogenic competence.

Regeneration recalcitrance in wheat is a complex, multigenic trait under both genetic and epigenetic control. Its manifestation reflects the convergence of limited dedifferentiation capacity, hormonal imbalance, stress-related metabolic constraints, and restrictive chromatin states, all of which reduce the ability of explant cells to re-enter embryogenic pathways. Limited dedifferentiation capacity has been associated with reduced expression of key morphogenetic regulators, including *WUS*, *BBM*, *LEC1/LEC2*, and *SERK*. Additionally, physiological constraints, such as hormonal imbalance between auxins and cytokinins, insufficient antioxidant activity, and excessive accumulation of reactive oxygen species (ROS) in callus tissues, contribute to impaired embryogenic potential and increased cellular mortality [37]. These responses predominantly reflect abiotic stress conditions inherent to in vitro culture, arising from artificial hormone regimes, oxidative stress, and nutrient limitations. In contrast, biotic stress, typically associated with a pathogen or pest attack, activates immune-related signaling pathways mediated by salicylic acid, jasmonic acid, and ethylene. Although biotic stress is largely absent under sterile culture conditions, both abiotic and biotic stress responses share common signaling components, including ROS signaling, MAP (Mitogen-activated protein kinase) kinase cascades, and transcriptional reprogramming, which may collectively reinforce the recalcitrant phenotype. These stress-related factors often interact synergistically with transcriptional and epigenetic restrictions, reinforcing the recalcitrant phenotype.

Epigenetic mechanisms also play a central role in determining regenerative competence. DNA methylation patterns, histone modifications, and chromatin accessibility influence the activation of developmental genes during dedifferentiation. Comparative analyses have revealed distinct epigenetic profiles between highly regenerable and recalcitrant genotypes, suggesting that regenerative capacity is closely linked to chromatin state and transcriptional plasticity [31,32].

Ultimately, dissecting the molecular and epigenetic foundations of recalcitrance and identifying early biomarkers of regenerative competence is essential for developing transformation systems that function reliably across elite wheat cultivars. Such advances are expected to accelerate the application of biotechnology and precision breeding in this economically important but technically challenging crop.

### 2.2. Differences Among Wheat Lines and Cultivars

Differences in regenerative capacity among wheat genotypes are well documented. Model cultivars such as ‘Bobwhite’ and ‘Fielder’ are considered highly transformable and exhibit enhanced regenerative competence, which is associated with elevated expression of morphogenetic genes, including *WUS*, *BBM*, and *SERK* [15,38]. Studies have shown that the ‘Fielder’ cultivar can achieve up to 90% transformation efficiency using an optimized *Agrobacterium*-mediated protocol, whereas other genotypes exhibit substantially lower success rates (<10%) [20,39]. Their responsiveness to standard tissue culture conditions has made them the primary platforms for studies on wheat transformation, functional genomics, and CRISPR-Cas-based editing. In contrast, elite cultivars with high agronomic value, such as ‘Kontesa’ and ‘Ostka Smolicka’, often display low capacity for callus induction and shoot regeneration.

Key differences among wheat lines arise from multiple factors, including the developmental stage of the embryos (with the highest competence observed between 0.8 and 1.5 mm in length), the composition of the culture medium, and the auxin-to-cytokinin ratio, as well as the metabolic and epigenetic activity of the explant. For the ‘Bobwhite’ cultivar, the use of immature embryos maintained for 1–6 days on CM4C medium provides the best regeneration results, whereas in ‘Fielder’, optimal outcomes are obtained using 9-day-old embryos [40]. These genotype-specific optima emphasize the absence of a universal regeneration system for wheat and highlight the need for tailored protocols, particularly when working with agronomically relevant cultivars.

### 2.3. Epigenetic and Metabolic Determinants of Recalcitrant Regeneration

Epigenetic regulation plays a central role in shaping wheat regenerative competence, as chromatin accessibility determines the ability of somatic cells to reinitiate totipotential developmental programs. Treatments with histone deacetylase (HDAC) inhibitors such as trichostatin A (TSA) significantly enhance regeneration efficiency, reflecting the importance of histone acetylation in maintaining an open chromatin state permissive for transcriptional reprogramming [41]. Recent epigenomic analyses further demonstrate that the repressive histone modification H3K27me3 is closely associated with embryogenesis and cell differentiation processes [42]. In addition to epigenetic regulation, cellular metabolism plays a crucial role in determining regenerative capacity. Wheat cells exposed to abiotic stress conditions inherent to in vitro culture, such as oxidative stress induced by artificial hormone treatments, light exposure, and nutrient imbalance, often exhibit excessive production of ROS, which disrupts redox homeostasis. Insufficient antioxidant capacity frequently impairs embryogenic initiation in wheat callus. Prevention of oxidative stress through supplementation of antioxidants, such as ascorbic acid or glutathione, or by short-term dark pre-incubation enhances viability and supports the transition toward somatic embryogenesis [43].

Another important factor is polyamine metabolism, which contributes to DNA stability, membrane integrity, and modulation of stress responses. Increasing the levels of spermidine and spermine in the culture medium promotes the formation of embryogenic structures and enhances callus viability [44]. Together, these epigenetic and metabolic constraints ultimately determine whether explant cells can acquire and sustain regenerative competence, a limitation that is particularly pronounced in inherently recalcitrant wheat genotypes.

## 3. Biology of Wheat Regeneration

In vitro regeneration of wheat is a complex process whose efficiency depends on multiple interacting factors, including genotype, explant age, culture medium composition, light conditions, and hormonal regulation. Both organogenesis and somatic embryogenesis play key roles in the transformation and propagation of elite wheat genotypes. Understanding the biology of these processes forms the foundation for optimizing regeneration systems and improving the efficiency of genome modification.

### 3.1. Types of Regeneration: Organogenesis and Somatic Embryogenesis

Organogenesis involves the formation of new organs (shoots and roots) either directly from explant tissues or indirectly through a callus phase. During this process, differentiated cells undergo dedifferentiation and subsequently reorganize into apical meristems capable of further development [45]. The balance between auxins and cytokinins plays a crucial role: high auxin levels promote root initiation, whereas cytokinin dominance induces shoot formation.

Somatic embryogenesis (SE), in contrast, refers to the development of somatic embryos from somatic cells independently of fertilization [46]. This process includes several stages of embryogenic induction, embryo development and maturation, germination, and regeneration of a complete plant. Each stage involves significant transcriptional and epigenetic reprogramming leading to the activation of key developmental genes, such as *SERK*, *WUS*, *BBM*, *LEC1*, *LEC2*, and *FUS3* [47].

In wheat, somatic embryogenesis is considered to be a more fundamental type of regeneration, as it involves the re-creation of the entire embryonic structure from a single cell. Unlike organogenesis, SE results in progeny with high genetic stability and reduced somaclonal variation [45]. In the context of wheat, somatic embryogenesis is of particular importance, as it enables the transformation and regeneration of recalcitrant genotypes. High SE efficiency is a critical determinant of *Agrobacterium*-mediated transformation and genome-editing protocols employing the CRISPR-Cas9 system [14,48].

### 3.2. The Cellular Competence Window—Embryo Stage and Explant Type

One of the most important factors determining the success of in vitro wheat regeneration is the so-called “cellular competence window”—the developmental phase during which explant cells exhibit the highest potential for dedifferentiation and embryogenesis. In wheat, the optimal explant material consists of immature embryos at the milk stage, ranging from 0.8 to 1.5 mm in length. Larger embryos (>2 mm) display reduced embryogenic potential and lower regeneration frequencies, as they rapidly undergo differentiation [40].

Although immature embryos remain the preferred explant type in most transformation systems, alternative tissues such as coleoptile segments, hypocotyl fragments, or pre-induced embryogenic callus can also be used. However, these explants differ considerably in hormonal responsiveness and developmental plasticity, resulting in substantial variation in regeneration frequency across genotypes and protocols [49]. In addition, both methodology reviews and recent experimental work demonstrate that mature embryos can also serve as suitable explants: either indirectly, via embryogenic callus derived from mature seed tissues, or directly, through *Agrobacterium*-mediated transformation of mechanically isolated mature embryos followed by regeneration through organogenesis [40,49].

The systems based on mature embryos exploit the long-term storability and year-round availability of dry seeds, improving scalability, flexibility, and experimental consistency, although in many genotypes their regeneration efficiency still tends to be lower than that of optimally staged immature embryos [40,49].

Taken together, the concept of a cellular competence window highlights that successful regeneration depends on initiating cultures from tissues in which the chromatin state, hormonal sensitivity, and developmental programming are still permissive for the activation of totipotential pathways. This principle underpins the design of efficient transformation systems and explains the consistently superior performance of very young wheat embryos compared with more differentiated tissues.

### 3.3. The Role of Meristematic Tissues and Embryogenic Callus

The formation of embryogenic callus represents a pivotal transition in wheat regeneration, marking the point when somatic cells regain meristematic identity and the capacity to initiate organogenic or embryogenic development. Embryogenic callus represents the key intermediate stage between somatic cell dedifferentiation and somatic embryo formation. It develops as a result of structural reorganization of the cells and activation of genes responsible for maintaining pluripotency. Transcription factors from the *WOX*, *BBM*, *SERK*, and *LEC* families play pivotal roles in this process. Activation of *WUS* reestablishes meristematic cell identity, while *BBM* initiates the transition toward embryogenesis [30].

Histological analyses of embryogenic callus in wheat reveal the presence of small cells with dense cytoplasm undergoing active division, whereas enzymatic studies indicate elevated peroxidase activity and enhanced function of oxidative stress reduction pathways in these tissues [50,51].

Callus formation is regulated mainly by plant hormones. Auxins such as 2,4-D induce dedifferentiation and callus induction, whereas cytokinins (e.g., BA (benzyl adenine), kinetin) stimulate shoot formation. The key transition from callus to organogenesis involves the activation of *ARR* and *WUS* genes, which control the initiation of shoot meristems [6,52,53].

### 3.4. Environmental Factors

In addition to genetic and hormonal factors, environmental conditions such as light, temperature, and nutrient composition strongly influence in vitro regeneration. Light exposure plays a dual and stage-dependent role during in vitro culture. While light is essential for chloroplast differentiation and shoot development at later stages, exposure during the early phases of explant culture and callus induction can exacerbate photooxidative stress. Excessive light promotes the accumulation of ROS, which disrupt cellular redox balance and interfere with auxin-mediated signaling required for embryogenic reprogramming. Studies in *Arabidopsis thaliana* have demonstrated that short-term dark incubation (2–24 h) following explant excision significantly enhances shoot regeneration frequency by reducing ROS levels and stabilizing the hormonal gradient [54]. A similar phenomenon has been observed in wheat: explants maintained under limited light during the callus induction phase exhibit higher viability and greater numbers of embryogenic structures. Temperature also plays a decisive role. Optimal embryogenesis in wheat is generally achieved at temperatures between 24 and 26 °C, which support balanced enzymatic activity, efficient energy metabolism, and proper cell-cycle progression. Deviations from this optimal range induce physiological stress: low temperatures (<18 °C) slow metabolic processes and delay cell division, whereas elevated temperatures (>30 °C) accelerate respiration, enhance ROS production, and destabilize cellular membranes. These temperature-induced stresses impair somatic embryo initiation and development, ultimately reducing regeneration efficiency [55,56,57].

### 3.5. The Role of Embryogenesis in Wheat Genetic Engineering

Somatic embryogenesis is not only a model for studying plant totipotency but also a central component of wheat genetic engineering. In CRISPR-Cas-based editing systems used for transformation, the regeneration phase determines whether edited or transgenic cells can develop into stable, fertile plants capable of transmitting genetic modifications to the next generation [58]. Therefore, the efficiency of embryogenesis directly influences the number of successful editing events, the proportion of non-chimeric regenerants, and the overall speed at which genome-modified lines are created.

From a practical perspective, high embryogenic capacity enables the rapid expansion of individual edited cells into uniform embryogenic structures, greatly reducing mosaicism and increasing the likelihood of producing plants carrying fully fixed edits [30]. This is particularly important in wheat, where transformation frequencies are often low and strongly genotype-dependent, making each successfully regenerated embryo a valuable specimen of edited material; as has been repeatedly emphasized, regeneration efficiency is the major limiting factor in both transgenic and genome-editing approaches [38].

We therefore argue that optimization of somatic embryogenesis is not an auxiliary improvement but a prerequisite for effective wheat genome engineering. This requirement becomes especially pronounced in multiplex CRISPR editing strategies and in efforts to introduce targeted alleles into elite cultivars, which often exhibit inherently poor regenerative responses [59,60]. Addressing embryogenesis-related constraints is thus essential for translating genome-editing technologies into practical wheat improvement programs.

## 4. Signaling Peptides and Receptors

Signaling peptides and their receptors are key components of the regulatory networks that control tissue renewal and differentiation in plants. In wheat, the CLE/CLV-WUS, PSK/PSKR, and SERK/BRI1 systems are functionally interconnected and act together in the regulation of meristems and somatic embryogenesis. Their modulation, either at the level of gene expression or through the addition of exogenous ligands, can significantly enhance the regenerative competence of wheat in vitro.

Table 1 summarizes major signaling peptide families and receptor-like kinases implicated in the control of tissue competence and somatic embryogenesis.

### 4.1. CLE/CLV–WUS Pathway and Meristem Control

The *CLE*/CLV-WUS (CLAVATA-WUSCHEL) signaling pathway is one of the best-characterized mechanisms controlling stem cell activity in plant shoot and root meristems. It functions as a feedback loop between the peptide ligand CLE (CLAVATA3/ESR-related) and the transcription factor WUS, which maintains cells in a pluripotent state [63]. High concentrations of CLE ligands activate the CLAVATA1/CLAVATA2 (CLV1/CLV2) receptor complex, which, through a kinase cascade, suppresses *WUS* expression and thereby limits meristematic cell proliferation. Conversely, when the CLE signal is weak or absent, *WUS* expression is reactivated, expanding the meristematic domain [61].

Comparative studies have shown that wheat has conserved orthologs of this pathway, including *TaCLV1*, *TaCLV2*, and *TaWUS2*, with functions analogous to their *Arabidopsis thaliana* counterparts [72,73]. Overexpression of *TaWUS2* increases the number of embryogenic calli and improves somatic embryo regeneration, confirming the significance of this signaling route in cereal regeneration processes.

Experimental application of synthetic CLE ligands (e.g., CLV3/CLE peptide) has demonstrated that controlled modulation of this system can influence meristem size and thus regulate embryogenic activity. High doses of CLE ligands inhibit regeneration, whereas their reduction or blocking of the CLV1 receptor promotes *WUS* reactivation [62].

In wheat biotechnology, understanding the dynamics of the *CLE*/CLV–WUS pathway has important practical implications for improving in vitro regeneration and genetic transformation efficiency. Precise manipulation of this regulatory module enables the enhancement of cellular competence for regeneration, for example, through transient or inducible expression of *WUS* to promote stem cell identity and embryogenic reprogramming during early culture stages. Conversely, partial inhibition of *WUS*’s negative regulation mediated by CLV signaling components can prolong meristematic activity without inducing developmental defects when applied in a spatially or temporally controlled manner. Such strategies facilitate somatic embryogenesis, increase transformation success rates, and provide a promising approach to overcoming genotype-dependent recalcitrance in wheat tissue culture systems [52,61,63].

### 4.2. PSK (Phytosulfokine) as a Proliferation-Stimulating Peptide

Another important signaling peptide involved in growth and regeneration regulation is PSK (phytosulfokine), a pentapeptide with the amino acid sequence YIYTQ, first identified in carrot cell cultures [74]. PSK acts through membrane-bound receptors PSKR1 and PSKR2, which belong to the LRR-RLK (Leucine-Rich Repeat Receptor-Like Kinase) family. Upon receptor binding, PSK activates a MAPK signaling cascade that stimulates cell division and upregulates genes associated with the cell cycle [65].

In cereals, the exogenous addition of PSK to culture media has been shown to increase callus biomass and the number of somatic embryo-like structures. In wheat, overexpression of *TaPSK2* correlates with higher in vitro regeneration efficiency and a greater number of regenerated shoots [64]. This mechanism is attributed to the activation of genes that maintain cell proliferation and delay callus cell differentiation.

Furthermore, PSK enhances cellular tolerance to oxidative stress, indirectly supporting the embryogenic process. It is currently one of the few signaling peptides with practical applications in plant biotechnology. Its incorporation into regeneration protocols allows for production of more stable and uniform embryogenic callus populations.

### 4.3. SERK as a Marker of Somatic Embryogenesis

The *SERK* gene family encodes membrane-associated coreceptors involved in the initiation of somatic embryogenesis and in hormone signaling, including the brassinosteroid pathway [75]. Under in vitro conditions *SERK1* and *SERK2* expression rises sharply in cells undergoing dedifferentiation.

In wheat, *TaSERK1* and *TaSERK2* achieve high expression levels in embryogenic callus tissues, and their overexpression correlates with increased numbers of embryonic structures [69,76,77]. SERK proteins act as coreceptors in brassinosteroid signaling through interaction with LRR-RLK receptors. Activation of the SERK-BRI1 complex triggers signal transduction pathways that lead to cell-cycle reactivation and growth.

Beyond their role in developmental regulation, SERK1 and SERK2 also participate in defense-related signaling pathways by acting as coreceptors in ligand-induced complexes formed after perception of pathogen-associated molecular patterns. Although they do not function as canonical resistance genes, SERK proteins contribute to early stress- and defense-associated signaling and act redundantly with other SERK family members, particularly BAK1 [77]. This multifunctionality highlights the role of SERK proteins as signaling hubs that integrate developmental and stress-related cues.

Moreover, SERK contributes to the formation of a microenvironment favorable for embryogenesis by modulating hormonal gradients within the callus [78]. For this reason, SERK is widely used as a marker of somatic embryogenesis; its expression can identify regeneration-competent cells even before visible embryonic structures appear.

In wheat biotechnology systems, monitoring *TaSERK1/2* transcript levels is used to assess callus quality and optimize culture conditions. Enhancing SERK signaling, by either hormone therapy or targeted gene expression, is a way to improve regeneration efficiency, especially in recalcitrant genotypes.

## 5. Pro-Regenerative Transcription Factors (Morphogens)

Transcription factors play a central role in regulating dedifferentiation, embryogenesis, and in vitro regeneration. They are referred to as molecular morphogens because their controlled expression is sufficient to reprogram cell identity and trigger embryogenic pathways independently of hormonal cues. In wheat, numerous genes homologous to well-known regulators from model species, such as *Arabidopsis thaliana*, maize, and rice, have been identified. Among these, *WUS*, *BBM*, *SERK*, *GRF*-*GIF*, *LEC1*/*LEC2*, *WOX5*, *PLT*, *LAX1*, and *WIND1*/*ERF115* are of particular significance.

These transcription factors function as molecular switches of plant developmental programs: their spatial or temporal expression can be sufficient to initiate embryogenic or organogenic development. The following sections summarize the major pro-regenerative factors, emphasizing their mechanisms, interactions with hormonal signaling, and experimental evidence, particularly in cereals. Transcription factors such as WUS, BBM, GRF-GIF, LEC, WOX, and SERK act as molecular regulators of dedifferentiation and tissue renewal in wheat. Their application in genetic transformation systems enables the overcoming of recalcitrance barriers and markedly enhances in vitro regeneration efficiency. The combined use of *WUS2*/*BBM* or *GRF*-*GIF* is currently regarded as one of the most promising strategies for optimizing wheat and other cereal transformation protocols.

Together, pro-regenerative transcription factors redefine the biological limits of wheat regeneration, providing a conceptual and practical framework for designing efficient transformation and genome-editing systems.

**Figure 1:**
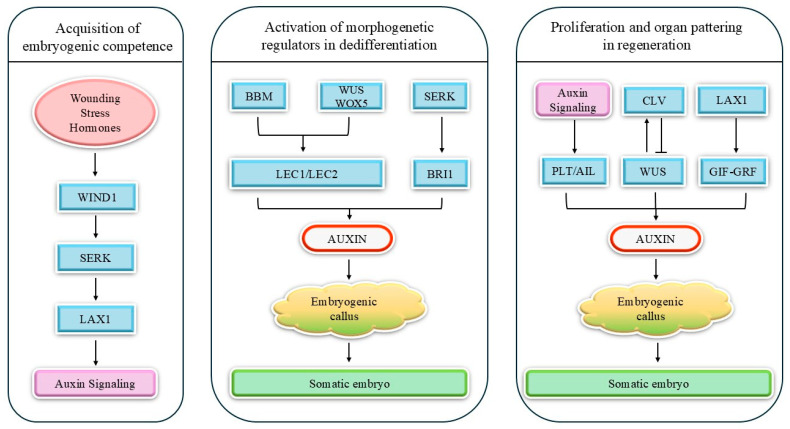
Regulatory network of pro-regenerative transcription factors controlling somatic embryogenesis and regeneration.

Figure 1 summarizes the functional organization of pro-regenerative transcription factors that control somatic embryogenesis and regeneration in cereals. In the first phase, wounding, stress, and exogenous hormones induce *WIND1/ERF115*, which upregulates *SERK* and the auxin transporter *LAX1*, thereby enhancing auxin signaling and conferring embryogenic competence to responsive cells.

In the dedifferentiation phase, BBM, together with stem cell regulators WUS and WOX5, activates LEC1/LEC2, while SERK and BRI1-dependent signaling further stabilizes the embryogenic state. This regulatory module promotes auxin biosynthesis/sensitivity, leading to the formation of embryogenic callus and de novo somatic embryos.

During regeneration, auxin signaling activates PLT/AIL factors that sustain proliferative capacity and root pole establishment, whereas WUS, modulated by the CLV pathway, together with LAX1-mediated auxin transport and the GRF-GIF complex, promotes shoot meristem activity and organ patterning. The scheme thus integrates WIND1/ERF115, WUS/WUS2, BBM, SERK, GRF-GIF, LEC1/LEC2, WOX5, PLT, and LAX1 into a coherent network of molecular morphogens that underpins enhanced wheat transformation and regeneration.

### 5.1. BBM—Somatic Embryogenesis

The *BBM* gene belongs to the *AP2*/*ERF* transcription factor family and represents one of the strongest known inducers of somatic embryogenesis. It was first identified in *Brassica napus*, and its orthologs are present in most crop species [79].

*BBM* activates embryogenic programs by inducing the expression of *LEC1*, *LEC2*, *FUS3*, and *ABI3* genes, which control the early stages of somatic embryo formation. Furthermore, it influences the expression of genes involved in auxin biosynthesis and transport, promoting the local accumulation of IAA necessary for embryogenesis initiation.

Under in vitro conditions, *BBM* overexpression leads to the spontaneous formation of embryogenic structures even in the absence of exogenous auxins, confirming its role as a morphogen essential for the dedifferentiation of somatic cells [79].

*BBM* strongly interacts with hormonal pathways; the activation of its expression increases transcript levels of *YUC* genes responsible for auxin biosynthesis and may cooperate with cytokinin-dependent regulators during later organogenic stages. In cereal studies, the *BBM* gene has been applied in transformation systems with increased regeneration efficiency. In wheat, introduction of a construct containing the *ZmBBM* gene (originating from maize) under the control of an inducible promoter resulted in several-fold increases in the number of regenerating calli [30]. In another study, a combination of *BBM* with *WUS2* achieved over 70% regeneration efficiency in genotypes previously considered recalcitrant. These results confirm that simultaneous activation of embryogenic factors and meristematic regulators significantly enhances the regenerative competence of cells [80].

From a practical perspective, BBM is now used in “genetically assisted regeneration systems”, where its expression is transiently activated in transformed cells and subsequently silenced after plant regeneration (e.g., via the Cre-loxP recombination system). This approach minimizes pleiotropic effects of *BBM* overexpression while markedly improving transformation and regeneration efficiency, particularly in cereals and other monocot species [30,80,81].

Supplementary Figure S1 illustrates the evolutionary framework of the analyzed genes by depicting phylogenetic relationships among cereal species and the degree of coding-sequence conservation. This context is particularly important because coding sequences derived from different species are frequently employed in wheat transformation systems. A notable example is the use of *BBM* and *WUS2* genes originating from maize, which have been shown to significantly enhance regeneration efficiency when introduced into wheat [30].

### 5.2. GRF-GIF Complex—miR396 Regulation and Enhancement of Regenerative Competence

The *GRF-GIF* (*Growth-Regulating Factor-GRF Interacting Factor*) complex represents one of the most recent and promising discoveries for improving regeneration and transformation of crop plants, particularly cereals.

GRF is a family of transcription factors whose activity is precisely regulated by the microRNA miR396. High levels of this microRNA limit GRF expression, thereby restricting cell growth and reducing embryogenic and regenerative potential [38,82].

GIF acts as a transcriptional cofactor and forms a stable complex with GRF, enhancing its transcriptional activity and protein stability [83]. The mechanism of the GRF-GIF complex activity involves reactivation of cell divisions in explant cells, upregulation of cell-cycle genes (e.g., *CYCD3;1*, *CDKB2;1*), and maintenance of cells in a proliferative state for an extended period [84].

Fusion of *GRF4-GIF1* genes combines the activity of both components into a single protein, thereby eliminating negative regulation by miR396 and stabilizing the transcriptional effect, leading to a significant increase in tissue regeneration [38].

In wheat, the use of a *TaGRF4-TaGIF1* construct resulted in a several-fold increase in the number of regenerated shoots and transformation efficiency. Similar results were obtained in rice, maize, and sorghum using *OsGRF4-OsGIF1* and *ZmGRF5-ZmGIF1* complexes, demonstrating a high degree of conservation among monocot species [30].

The GRF-GIF complex is also linked to hormonal regulation. Activation of its components increases cellular sensitivity to cytokinins and auxins, allowing for regeneration even at lower concentrations of exogenous hormones. Expression of GRF-GIF activates auxin biosynthesis genes (*YUC*) and cytokinin signaling pathways (*ARR*), promoting embryogenic callus induction and shoot development [38].

From a practical perspective, *GRF*-*GIF* chimeras are now used in genetically assisted regeneration systems, enabling effective transformation even in recalcitrant genotypes. Combining *GRF-GIF* constructs with CRISPR-Cas9 technology has yielded numerous regenerated and genome-edited wheat plants, including those with edits in the *Q* gene responsible for grain traits [38].

Supplementary Figure S2 presents the phylogenetic relationships of *GIF* genes across cereal species and highlights the conservation of their coding sequences. This analysis is relevant because GIF proteins act as conserved cofactors of GRFs, allowing heterologous *GIF* genes from related species to be considered for use in wheat transformation systems to support cell proliferation and regeneration.

Supplementary Figure S3 illustrates the evolutionary relationships of GRF genes among cereal species, together with the degree of coding-sequence conservation. Given the central role of GRFs in controlling cell proliferation and growth, their high evolutionary conservation supports the use of GRF genes from other cereals to enhance regeneration efficiency in wheat transformation.

### 5.3. LAX1—Regulation of Lateral Organ Formation and Meristematic Competence

The *LAX1* (*LAX PANICLE 1*) gene encodes a basic helix–loop–helix (bHLH) transcription factor crucial for the initiation and maintenance of axillary meristems (AMs) in grasses, including panicle and spikelet development. In rice (*Oryza sativa*), *LAX1* is essential for axillary meristem formation [85,86].

In the context of in vitro regeneration, its significance lies in maintaining the activity of shoot meristematic cells and the formation of lateral buds, theoretically contributing to increased regeneration in explant cultures. In rice, *LAX1* mutations lead to severely reduced ability to form lateral meristems and fewer lateral shoots, suggesting that the wheat ortholog *TaLAX1* might perform a similar function [87].

Inducing *LAX1* expression in regeneration programs could improve the balance between proliferation and differentiation of meristematic cells, resulting in a higher number of regenerating shoots in callus cultures.

The mechanism of LAX1 action involves early activation at specific leaf stages (e.g., plastochron P4) in the region between the developing leaf and the initiating lateral meristem. In this zone, *LAX1* mRNA and protein accumulate, and the protein can move into cells initiating the lateral meristem, enabling their proliferation and transformation into meristematic structures [85,86].

Additionally, LAX1 integrates developmental and hormonal signals, particularly auxin gradients and local cytokinin changes, that define the meristem initiation zone. Disruption of LAX1 function reduces lateral bud formation and alters plant architecture, indirectly affecting regenerative competence.

Although there are fewer direct studies on *TaLAX1* in wheat compared to the studies of the *LAX1* gene in rice, the high conservation of this mechanism among grasses suggests that manipulating *LAX1* orthologs could improve bud formation in vitro and enhance organogenesis success [86,87].

The phylogenetic analysis shown in Supplementary Figure S4 depicts the relationships and coding-sequence conservation of *LAX1* genes across cereals. As LAX1 is involved in auxin transport and organogenesis, the conserved nature of these genes suggests that orthologs from other species may be functionally applicable in wheat regeneration and transformation studies.

### 5.4. SERK—Participation in Embryogenesis and Hormonal Signaling

The SERK family proteins function not only as membrane receptors but also as regulators of embryogenic gene expression. SERK participates in brassinosteroid signaling and in activating somatic embryogenesis pathways, acting as a coreceptor in receptor complexes and transducing signals that modulate the expression of embryogenic and cell-cycle genes [75,77,78].

In wheat, overexpression of *TaSERK1* and *TaSERK2* increased the number of somatic embryos and improved regeneration, while *SERK* expression is often coregulated with *BBM* and *WUS*, indicating synergistic action [76,77]. High SERK levels are observed in cells initiating embryogenesis, with expression decreasing during later differentiation stages [69,78].

In wheat, the families *TaSERK1/2/3* demonstrate inducible expression in callus cultures [77]. SERK operates in conjunction with brassinosteroid signaling through the SERK-BRI1 complex, influencing proliferation and growth gene expression. Additionally, *TaSERK* is auxin-inducible in cell cultures, directly linking it to dedifferentiation mediators [37,76,77].

*TaSERK* expression correlates with embryogenic competence, and its use as a marker allows for an early identification of promising calli. Manipulating *SERK* signaling, e.g., via brassinosteroid application, can increase somatic embryo formation [76,77].

Supplementary Figure S5 shows the phylogenetic framework of *SERK* genes and the conservation of their coding regions among cereal species. *SERK* genes are key regulators of somatic embryogenesis and cellular competence, and their evolutionary conservation supports the use of heterologous *SERK* genes to improve callus induction and regeneration in wheat.

### 5.5. WIND1/ERF115—Wound-Induced Dedifferentiation and Regeneration

*WIND1* (*WOUND INDUCED DEDIFFERENTIATION1*) and *ERF115* belong to the *AP2/ERF* family and play key roles in plant wound response, triggering dedifferentiation and regeneration processes [88,89,90].

After mechanical wounding, *WIND1* expression rapidly increases in cells surrounding the injury, leading to dedifferentiation and callus formation [89,91]. ERF115 activates divisions of meristematic cells and supports shoot regeneration in response to wounding, partly through interactions with locally accumulating auxin [88,92].

In wheat, orthologs such as *TaERF115* and *TaWIND1* are highly expressed in embryogenic callus, suggesting their potential role in maintaining regenerative competence in in vitro cultures. Incorporating *WIND1/ERF115* overexpression into transformation systems may increase the number of induced calli, particularly in wounded explant cultures. This mechanism relies on reactivating cellular programs normally active during embryogenesis or organogenesis, allowing for regeneration even with limited exogenous hormones [88,90].

WIND1 acts as a “switch,” triggering a wide transcriptional cascade that includes genes related to chromatin remodeling, the cell cycle, and meristematic expression. Its activity integrates mechanical, hormonal (auxin, cytokinin, jasmonate), and epigenetic signals [88,91].

Induction of *WIND1* occurs upon wounding, independent of added auxins or cytokinins, but its function aligns with local auxin and cytokinin signaling changes at the wound site. Importantly, WIND1-mediated wound responses are accompanied by epigenetic reprogramming, including derepression of Polycomb Repressive Complex 2 (PRC2) target loci, which leads to reduced H3K27me3 levels at genes associated with cell-cycle re-entry, dedifferentiation, and meristematic identity. This chromatin remodeling facilitates the activation of regeneration-associated transcriptional programs, thereby enabling callus formation and tissue repair following injury [88,91].

In *Arabidopsis thaliana*, *WIND1* overexpression led to dedifferentiation even without exogenous hormones, making it a promising tool for in vitro regeneration protocols [90,92]. Although direct studies on wheat (*TaWIND1-TaERF115*) remain limited, the high conservation of this mechanism in higher plants suggests its potential utility in improving cereal regeneration systems.

Supplementary Figure S6 summarizes the evolutionary relationships of *WIND1* genes and the conservation of their coding sequences in cereals. As *WIND1* plays an important role in wound-induced dedifferentiation, conserved *WIND1* orthologs may contribute to the acquisition of regenerative competence in wheat tissue culture systems.

### 5.6. WOX5—Regulation of Root Stem Cell Identity

The *WOX5* (*WUSCHEL-RELATED HOMEOBOX 5*) gene, belonging to the same family as *WUS*, regulates stem cell identity in the root meristem [93]. In wheat, its homolog *TaWOX5* is highly expressed during the initial stages of somatic embryogenesis and is gradually downregulated during the differentiation phase [94]. Induction of *WOX5* overexpression in explants increases the number of callus-initiating cells and accelerates their divisions, resulting in higher regeneration efficiency [94,95].

WOX5 functions within the root quiescent center (QC), balancing cell division and differentiation [96]. It operates downstream of auxin and cytokinin gradients that establish meristem organization. Manipulating *WOX5* expression can accelerate root meristem formation in regenerants, improving rooting efficiency in wheat transformation systems [97].

As a regulatory factor, WOX5 plays a central role in the root stem cell niche (the quiescent center, QC), maintaining the identity of columella cells and controlling the balance between cell division and differentiation [93,96]. In the context of somatic embryogenesis, WOX5 may support the establishment and maintenance of local root meristems in regenerants [95].

WOX5 interacts with auxin and cytokinin signaling within the meristem: auxin reinforces the gradients that stabilize *WOX5* expression in the QC, whereas cytokinins modulate its activity during transitions between proliferation and differentiation [96,98]. Manipulating *WOX5* expression can accelerate the formation of root structures in regenerants, which is advantageous in protocols where rapid rooting is desired. In wheat, reports on *TaWOX5* indicate increased expression during early embryogenic stages and improved transformation efficiency [94,97].

The phylogenetic analysis in Supplementary Figure S7 highlights the conservation of *WOX5* genes, particularly within the homeobox domain, across cereal species. This conservation is consistent with the conserved role of WOX5 in maintaining stem cell niches and supports its potential application in heterologous expression studies aimed at improving wheat regeneration.

### 5.7. WUS2/WUSCHEL—Meristem Maintenance and Shoot Induction

WUSCHEL (WUS) is a key regulator maintaining the pluripotency of meristematic cells. Belonging to the *WOX* family, it acts as a positive regulator of shoot meristem renewal. In wheat, its homolog *TaWUS2* has a similar function, and its overexpression significantly enhances callus regeneration [99].

In transformation systems using *BBM*, combining *WUS2* with *BBM* resulted in up to a fivefold increase in regenerated shoots [100]. Controlled *WUS2* expression, temporally or spatially limited, is critical, as its excessive activity can lead to abnormal tissue growth and failure to form mature organs. WUS2/WUSCHEL acts as a central regulator of meristem domain maintenance: WUS maintains pluripotency within the shoot apical meristem. *ZmWUS2* from maize, combined with *BBM*, functions synergistically: *WUS* sustains the meristematic cell pool, while *BBM* activates the embryogenic program [101].

Temporally restricted *WUS2* expression can mimic natural recruitment of cells into the meristematic domain, stimulating meristem and embryonic structure formation [102]. Hormonally, WUS influences cytokinin response regulation and tissue sensitivity to cytokinins, acting in coordination with cytokinin during shoot formation from callus. The *WUS-CLE/CLV* feedback loop regulates meristem domain size [101,102].

Practically, *WUS2-BBM* systems have achieved high transformation and regeneration efficiencies in maize. *WUS2* is also used in CRISPR systems to enhance editing efficiency, while controlled removal of expression cassettes post-regeneration eliminates morphogenic transgenes from the final lines [103].

Supplementary Figure S8 illustrates the phylogenetic relationships and high coding-sequence conservation of *WUS2* genes among cereals. This is particularly relevant because maize-derived *WUS2* is widely used in wheat transformation, where its heterologous expression markedly enhances regeneration efficiency by promoting meristematic cell identity.

### 5.8. LEC1/LEC2/FUS3—Activation of Embryogenic Programs

The *LEC1*, *LEC2*, and *FUS3* genes belong to the NF-YA and B3 families, and they form the core of somatic embryogenesis regulation in plants. In *Arabidopsis thaliana*, LEC1 and LEC2 induce the conversion of somatic cells into embryogenic cells and activate the expression of genes involved in storage protein and lipid biosynthesis, thereby supporting embryo maturation [104,105].

Wheat orthologs, such as *TaLEC1* and *TaLEC2*, have similar expression profiles in embryogenic cultures, indicating conservation of regulatory function in in vitro somatic embryogenesis [42,106]. Combining *LEC2* with *BBM* is particularly effective in initiating embryogenesis in protoplast cultures, suggesting a synergistic mechanism [107,108].

*LEC1/LEC2/FUS3* form the core of the somatic embryogenesis program: their activation induces embryonic gene expression, accumulation of storage compounds (proteins and lipids), and establishment of embryonic identity in somatic cells. In *lec* mutants, the somatic embryogenesis capacity is greatly reduced [81,104]. Hormonal regulation integrates auxin and abscisic acid (ABA) signals, crucial for embryo maturation and accumulation of storage substances [106,109].

From a practical perspective, *LEC* gene overexpression in model plants induces embryogenic traits, and in in vitro regeneration protocols, combinations such as *BBM* + *LEC2* or *LEC* manipulations greatly enhance somatic embryo formation, particularly in protoplast cultures [70,104].

Supplementary Figure S9 presents the evolutionary relationships of *LEC1* genes and their coding-sequence conservation across cereal species. As LEC1 is a key regulator of embryogenesis and developmental reprogramming, its conserved function supports the use of heterologous *LEC1* genes in wheat regeneration and transformation approaches.

### 5.9. PLT/AIL—Regulation of Root-Initiating Cell Identity

Genes from the *PLETHORA* (*PLT*) family and the related *AINTEGUMENTA-LIKE* (*AIL/ANT-like*) group belong to the *AP2*/*AIL* transcription factor family and play a crucial role in establishing and maintaining the identity of root-initiating cells, as well as regulating their proliferation [110,111].

In the model plant *Arabidopsis thaliana*, PLT1 and PLT2 are responsible for maintaining the stem cell niche within the root meristem, and their expression depends on the auxin gradient, which controls the organization of the root axis and defines the boundaries of cell proliferation [110,111].

AIL factors promote the proliferation of organ-initiating cells and act synergistically with PLT, supporting the establishment of meristems in regenerants [29]. Although there are no direct publications describing PLT1/2 activity in embryogenic wheat callus, it has been shown that *PLT* orthologs in cereals are highly conserved regarding their regulatory functions in root and embryogenic meristems [95].

Based on translational studies, it can be assumed that in vitro culture induces the overexpression of these genes and the formation of root structures and improves the proportion of regenerating plants. The mechanism of PLT/AIL action involves activating a network of genes controlling proliferation and the maintenance of meristematic competence. Auxin gradients induce *PLT* expression in the meristem zone, while cytokinins modulate the boundary between cell proliferation and differentiation [110,111].

During somatic embryogenesis, these genes support the establishment of local root meristems, which translates into more efficient shoot regeneration and faster rooting of regenerants [95].

The phylogenetic framework shown in Supplementary Figure S10 depicts the relationships and conservation of *PLT/AIL* family genes among cereals. These transcription factors regulate stem cell identity and developmental patterning, and their conserved sequences suggest that orthologs from other cereal species may be exploited to enhance regeneration capacity in wheat.

**Figure 2:**
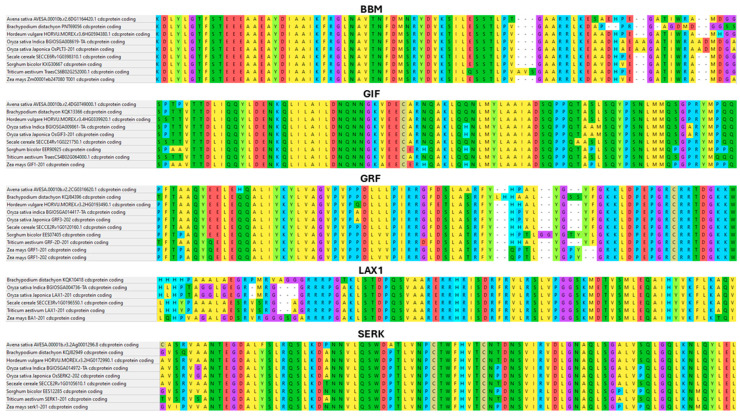

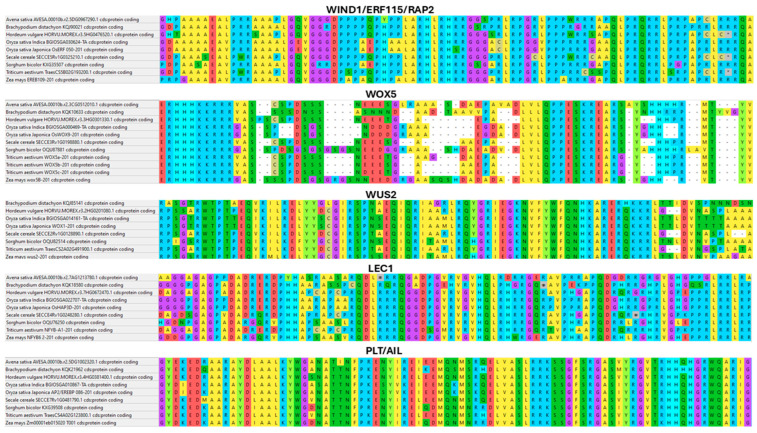
Multiple sequence alignment of key pro-regenerative regulators (BBM, GIF, GRF, LAX1, SERK, WIND1/ERF115/RAP2, WOX5, WUS2, LEC1, and PLT/AIL) from major cereal species.

The high evolutionary conservation of these morphogenic regulators across cereals is illustrated by the coding-sequence alignments shown in Figure 2. BBM, WUS/WUS2, WOX5, LEC1, PLT/AIL, GRF, GIF, LAX1, and SERK orthologs from wheat, barley, rye, oat, rice, maize, and sorghum share highly conserved protein regions, particularly within their DNA-binding and signaling domains (e.g., AP2/ERF domain in BBM, homeobox domains in WUS/WOX, B3 domain in LEC1, QLQ and WRC motifs in GRFs, LRR motifs in SERKs). In contrast, most sequence divergence is confined to low-complexity or intrinsically disordered segments thought to modulate species-specific regulatory interactions. This pattern supports the notion that the core molecular functions of these factors are conserved across cereals and helps to explain why heterologous combinations such as *ZmBBM-ZmWUS2*, as well as endogenous or homologous systems such as *TaGRF4-TaGIF1*, can efficiently reprogram wheat tissues and overcome genotype-dependent recalcitrance.

## 6. Strategies to Enhance Regeneration—Overview, Advantages, and Disadvantages

One of the key challenges in plant transformation is improving the regeneration of tissues into fully developed plants. This limitation arises from the restricted ability of somatic cells to re-enter totipotential developmental pathways under in vitro conditions. In recent years, considerable attention has been devoted to the use of morphogenic cassettes, also known as developmental regulators (DRs), whose expression enhances the competence of cells to transition from the callus stage to shoot and somatic embryo formation. The expression of regulators such as WUS, BBM, and GRF-GIF chimeras markedly increases the redifferentiation capacity of plant tissues. These morphogens act by transiently boosting cellular pluripotency and embryogenic competence, enabling somatic cells to respond more effectively to regeneration stimuli. This approach is particularly valuable in monocot species, which have historically been regarded as recalcitrant to transformation [30,38,112,113,114].

The general strategy involves short-term, controlled expression of morphogenic cassettes. Examples include *ZmWUS2* and *ZmBBM* in maize and the *TaGRF4-TaGIF1* chimera in wheat. Expression typically occurs during the callus induction phase, when cells exhibit the greatest developmental plasticity and the highest potential for reprogramming. At this stage, chromatin is more accessible and hormone sensitivity is elevated, making cells particularly receptive to developmental reactivation. It is essential to restrict expression of the developmental regulators to the early stages of regeneration, as this minimizes the adverse effects of sustained overexpression, such as altered meristem architecture, abnormal shoot morphology, and reduced fertility [29,112,113]. Restricting morphogen activity to a narrow developmental window preserves their regenerative benefits while preventing long-term disruptions to meristem organization.

Representative morphogenic regulators and their documented applications in wheat transformation and regeneration are summarized in Table 2.

After the initial induction of morphogenesis, morphogenic cassettes are usually removed using inducible Cre-loxP recombination systems, where the transgenes are flanked by *loxP* sites. This allows for excision of the cassette once somatic embryogenesis has been initiated, resulting in T_0_ plants that are fertile and free of morphogenic regulator genes. Such excision systems ensure that morphogen-driven reprogramming is strictly confined to early culture stages. An alternative approach is transgene segregation in the T_1_ generation. Both strategies enable recovery of plants devoid of morphogenic cassettes while retaining the benefit of enhanced regeneration [29,112].

Transient expression of WUS, BBM, and GRF-GIF provides several advantages. First, it increases the regenerative competence of cells in monocot species that traditionally have low transformation efficiency and strong genotype dependence. In maize, simultaneous expression of *ZmWUS2* and *ZmBBM* induces direct somatic embryogenesis, increasing the frequency of regenerated plants, even in elite or previously non-transformable lines. This is particularly important for elite wheat cultivars, which often fail to regenerate under standard tissue culture conditions [29,30,112,118].

Second, it shortens the transformation timeline. In optimized maize protocols, the time required to obtain transgenic plants has been reduced from approximately 16–22 weeks to only 7–10 weeks due to the use of *ZmBBM-ZmWUS2* cassettes and somatic embryogenesis-based regeneration instead of prolonged organogenesis [29,118].

Third, it significantly improves overall transformation efficiency. In maize, the *ZmWUS2-ZmBBM* combination not only enhances transformation frequency but also broadens the range of responsive genotypes [29,30,112]. In wheat, the *TaGRF4-TaGIF1* chimera greatly increased the proportion of regenerating plants in the ‘Fielder’ and ‘Bobwhite’ cultivars and enabled the recovery of CRISPR-edited plants even under reduced concentrations of exogenous growth regulators [38,114].

This platform offers additional opportunities for optimization. The choice of promoter (weak, stress-inducible, or tissue-specific), duration of expression, and dosage of the morphogenic regulator determine whether healthy shoots or abnormal structures are formed. Typically, a short expression window restricted to the callus phase is applied. Following recombinase-mediated cassette removal, T_0_ plants are obtained that are morphologically normal and fertile [29,112].

However, despite their effectiveness in promoting regeneration, the application of morphogenic regulators such as *WUS* and *BBM* is constrained by important biological limitations. Prolonged or uncontrolled overexpression of these genes can disrupt meristem organization, resulting in developmental defects, reduced fertility, and compromised progeny viability [29,112,113]. The threshold between beneficial and detrimental expression levels is narrow, and small deviations can strongly influence regeneration outcomes. Additionally, the need for cassette excision (via Cre-loxP) or subsequent segregation adds extra steps to the workflow. The efficiency of this strategy also remains genotype-dependent, and some cereal lines remain difficult to transform even when morphogenic regulators are employed. This likely reflects inherent differences in chromatin accessibility, hormonal response, and stress tolerance among cultivars. Therefore, individual optimization of promoter type, expression dosage, and timing remains essential [30,112,118].

In summary, transient expression of morphogenic cassettes such as *WUS*/*BBM* and *GRF*-*GIF* chimeras currently represents one of the most effective strategies for enhancing regeneration and genome-editing efficiency in crop species, particularly in monocotyledonous cereals such as maize and wheat. The success of this approach relies on precise temporal control of expression and subsequent cassette removal, which together enable the recovery of healthy, fertile T_0_ plants free from morphogenic regulator transgenes. As such, morphogen-assisted regeneration is becoming a foundational component of next-generation wheat transformation pipelines [29,30,38,112,114].

## 7. Examples of Applications in Transformation and Genome Editing

In recent years, advances in plant transformation and genome-editing technologies, particularly CRISPR-Cas9-based systems, have increasingly incorporated morphogenic factors to enhance regeneration efficiency, reduce chimerism, and shorten the time required to obtain T_0_ lines. This improvement largely results from the ability of morphogens to synchronize embryogenic development, allowing for a higher proportion of edited cells to contribute to regenerated shoots. Morphogenic regulators such as *WUS2* and *BBM* are used to induce somatic embryogenesis and improve tissue competence during transformation. In grasses, expression of *WUS2/BBM* has been shown to enhance regenerative capacity [119].

In the context of genome editing, combining morphogens with CRISPR-Cas9 not only improves overall transformation success but also increases editing efficiency. For example, in sorghum, the inclusion of *WUS2* significantly increased mutation frequencies and the number of gene-dropout events compared with conventional systems [119]. Morphogenic factors also help overcome strong genotype dependence by enabling efficient transformation of otherwise recalcitrant genotypes [118].

One of the major challenges in plant genome editing is chimerism—the presence of multiple genotypes within a single regenerated T_0_ plant—as well as the lengthy regeneration phase. The use of morphogens during transformation and genome editing shortens the period from somatic embryogenesis to shoot regeneration and T_0_ recovery. In the maize line ‘B104’, the use of *WUS/BBM* cassettes reduced tissue culture duration by approximately one month [118]. Similarly, in wheat (*Triticum aestivum*), the application of a morphogenic gene enabled completion of the transformation process in less than 90 days [115]. Morphogens are also associated with reduced chimerism due to more synchronized and efficient regeneration and produce a higher number of independent transformation/editing events per input unit of explant material [119].

In wheat, the model lines ‘Fielder’ and ‘Bobwhite’ were used to test the *TaGRF4-TaGIF1* chimera (also referred to as *GRF4-GIF1*). Transient expression of this fusion construct significantly enhanced shoot regeneration and overall transformation efficiency while maintaining normal T_0_ plant fertility [38,114]. Follow-up studies demonstrated that a miR396-resistant variant (*mTaGRF4*-*TaGIF1*) was also effective in more recalcitrant wheat genotypes such as ‘Kenong 199’, ‘Bobwhite’, and ‘Zhongmai 175’, yielding marked increases in regeneration frequency, higher CRISPR-Cas editing rates (including *TaALS* edits using a base editor), and earlier recovery of transgene-free plants [114].

In the maize inbred line ‘B104’, the morphogenic cassettes *ZmBBM*-*ZmWUS2* driven by tissue-specific promoters induced direct somatic embryogenesis from zygotic embryos. This substantially increased transformation efficiency and enabled integration of CRISPR-Cas9 modules targeting *loci* such as *VIRESCENT YELLOW-LIKE*, with heritable edits observed in the T_1_ generation [118]. Meanwhile, the use of controlled Cre-loxP recombination systems, including a heat-inducible *moCRE-loxP* module for excision of morphogenic genes after embryogenesis induction, mitigates negative effects of prolonged *BBM/WUS* expression and facilitates recovery of fertile T_0_ plants, as demonstrated in maize and sorghum [118,119].

The incorporation of morphogenic factors into plant transformation and genome-editing protocols represents a major step toward improving efficiency, reducing chimerism, and shortening the time required to obtain T_0_ lines. Literature examples demonstrate the practical effectiveness of this strategy, although careful control of morphogen expression and optimization of culture conditions and vector design remain essential to avoid phenotypic abnormalities. However, the regenerative boost provided by morphogens is tightly linked to precise temporal regulation, as excessive or prolonged activity can compromise plant development. In the future, the use of morphogens in transformation and editing workflows is expected to become a standard approach, particularly for crops that remain difficult to transform.

## 8. Summary and Perspective

Effective in vitro regeneration of wheat remains a prerequisite for both genetic transformation and advanced genome-editing strategies, including CRISPR-Cas9. This process is not merely a technical addition to the vector delivery stage; it determines whether individual modified cells can actually develop into fertile plants capable of transmitting the introduced changes and enabling their use in breeding. In wheat, one of the world’s key cereal crops, regeneration therefore represents the bottleneck of the entire biotechnological pipeline from vector construct to an improved breeding line with enhanced agronomic traits (yield, stress tolerance, grain quality). From a developmental perspective, regeneration requires activation of pluripotency and somatic embryogenesis programs, which involve profound transcriptional and epigenetic reprogramming of explant cells, as well as the reconstruction of functional meristems. In wheat, these processes are controlled by transcription factors from the WOX, PLT, BBM, ARR, and LBD families, which integrate auxin and cytokinin signals and redirect cells to a regenerative competence state [1,2,5,6,7].

Despite substantial technological progress, wheat remains a recalcitrant species with limited regenerative capacity. Regeneration efficiency is strongly genotype-dependent: model lines such as ‘Bobwhite’ and ‘Fielder’ are highly transformable and display elevated regenerative competence, partly due to favorable expression profiles of morphogenetic genes (*WUS*, *BBM*, *SERK*), whereas elite breeding cultivars—those of real agronomic relevance—often regenerate weakly, inconsistently, or not at all [14,15,16,20,39]. This discrepancy underscores that the biological foundations of regenerative competence differ substantially between model and elite cultivars.

In many valuable genotypes, transformation efficiency drops below 10%, whereas in ‘Fielder’, optimized *Agrobacterium*-mediated protocols have achieved efficiencies of up to ~90%.

These differences arise from multiple layers of regulation. First, the developmental stage of the explant is critical: the highest regenerative competence is observed in very young embryos of ~0.8–1.5 mm, whereas tissues exceeding ~2 mm rapidly differentiate and lose embryogenic potential. Second, the composition and hormonal balance of the culture medium, particularly the auxin-to-cytokinin ratio, play a decisive role in determining whether dedifferentiation or shoot initiation predominates. Third, the metabolic state of the tissue, including oxidative stress levels and the accumulation of reactive oxygen species, strongly influences cell viability and the ability to initiate somatic embryogenesis. Finally, the epigenetic background, such as repressive histone modifications associated with developmental silencing, further constrains the plasticity of explant cells [40,41,42,43].

Interestingly, environmental factors such as short-term dark incubation or antioxidant supplementation (e.g., ascorbic acid) can shift the redox balance toward a state more favorable for somatic embryogenesis and improve the survival of embryogenic callus.

In response to wheat’s inherent regenerative recalcitrance, molecular support strategies have been developed, based on the transient expression of so-called morphogens or developmental regulators (DRs). Key examples include *WUS*, *BBM*, members of the *SERK* family, and *GRF*-*GIF* chimeras (e.g., *TaGRF4-TaGIF1*). These morphogens act as developmental switches: their controlled expression is sufficient to induce cell dedifferentiation, initiate somatic embryogenesis, and stabilize new shoot or root meristems [30,38,114].

In wheat systems, the use of such factors has led to a reduction in T_0_ generation time (to less than 90 days), a decrease in chimerism among T_0_ plants, and an increase in the frequency of independent transformation and CRISPR-Cas9 editing events per unit of input tissue [115,119].

From a practical standpoint, an important finding is that *GRF*-*GIF* complexes, including *TaGRF4-TaGIF1* and its miR396-resistant variant, enhance regeneration not only in model lines (‘Fielder’, ‘Bobwhite’) but also in more challenging genotypes, such as ‘Kenong 199’ and ‘Zhongmai 175’, while maintaining T_0_ plant fertility [38,114].

This represents one of the first proofs that molecular support can genuinely unlock regeneration in agronomically important cultivars rather than only laboratory-friendly lines. It also indicates that the regenerative bottleneck in these cultivars is not absolute, but can be molecularly overcome under the right regulatory conditions.

Regulators such as SERK also serve as markers of embryogenic competence. In wheat, *TaSERK1* and *TaSERK2* are strongly induced in dedifferentiating cells, and their expression correlates with the ability of cells to form somatic embryos. In practice, this enables early assessment of callus quality before full regeneration and allows for optimization of culture conditions (e.g., using brassinosteroids to support the SERK-BRI1 pathway) [76,77,78].

Such early quality markers are particularly valuable in recalcitrant species, as they permit rapid selection of cultures with real regenerative potential, reducing the need for long and costly subcultures.

Experience from other grasses provides essential translational context. In maize (*Zea mays*), the co-expression of *ZmWUS2* and *ZmBBM* under tissue-specific promoters and restricted to early somatic embryogenesis dramatically increased transformation efficiency, shortened the time from infection to rooted plant recovery, and alleviated genotype barriers, making it possible to effectively transform lines previously considered non-transformable [30,80,118].

Moreover, combining morphogenic cassettes (*ZmBBM-ZmWUS2*) with CRISPR-Cas9 modules enabled precise editing of target *loci* with heritable mutations in the T_1_ generation [118].

A critical component of this strategy is developmental safety engineering: after embryogenesis initiation, *WUSBBM* cassettes are excised from the genome using Cre-loxP recombinase systems (including inducible moCRE-loxP systems, e.g., heat-triggered), preventing prolonged overexpression of these genes that would otherwise lead to meristem deformation and reduced fertility [29,112].

A similar approach was applied in sorghum, another historically recalcitrant species, confirming that “morphogen-assisted” regeneration is a general strategy for grasses, not merely a maize-specific innovation [119]. These cross-species outcomes demonstrate that the underlying developmental principles are conserved, making them directly informative for improving wheat regeneration.

The implication for wheat is that combining transient morphogen expression with subsequent DR cassette removal and concurrent genome editing could form a realistic working standard even for elite cultivars, not just model lines.

At the same time, several limitations of the current literature must be acknowledged. First, many reported successes in wheat regeneration and transformation still rely on model lines (‘Bobwhite’, ‘Fielder’), which are convenient for tissue culture but have limited breeding value [14,16]. Results obtained in these lines are often presented as best practices for wheat, even though transferring the same protocol to a commercial cultivar typically leads to an order-of-magnitude drop in efficiency or complete loss of regeneration.

Second, some studies report impressive numbers of T_0_ regenerants but rarely assess T_0_ fertility and T_1_ heritability, even though sustained overexpression of morphogens such as *WUS* or *BBM* is known to disrupt meristem organization and impair reproduction [29,112,113].

Third, definitions of regeneration and transformation efficiency remain inconsistent: some authors count the number of green shoots per callus, others the number of rooted plants per explant, and others the proportion of PCR-positive plants relative to total infections. The lack of a unified standard makes cross-laboratory comparisons nearly impossible and obscures whether a given technological innovation genuinely overcomes recalcitrance or merely optimizes an existing protocol for an easy line.

For these reasons, the discussion of progress in wheat regeneration would benefit from adopting a standardized reporting framework analogous to MIAPPE (Minimum Information About a Plant Phenotyping Experiment). MIAPPE was designed to enforce a minimal set of metadata in plant phenotyping studies, such as information about genetic material, environmental conditions, and measurement protocols. Adapting a similar philosophy for in vitro wheat culture could harmonize how regeneration and transformation experiments are described.

Practically, this would mean that each publication should at minimum report the precise genotype identity (model line vs. elite cultivar, breeding origin); explant age and size (e.g., immature embryo length in mm); complete medium composition, including auxin/cytokinin ratios and additives such as antioxidants, polyamines, or brassinosteroids; environmental conditions (photoperiod/darkness, temperature); morphogenic cassette details with promoters, expression window, and removal method (e.g., moCRE-loxP); and clear definitions of “regeneration efficiency” and “transformation efficiency,” including T_0_ fertility and T_1_ heritability.

The data clearly demonstrate that all these factors critically influence regeneration success, from embryonic developmental stage and oxidative stress control, through activation of *WUS*/*BBM*/*GRF-GIF* pathways, to subsequent DR cassette excision to avoid meristem disruption and fertility loss.

Implementing a MIAPPE-like standard for wheat tissue culture would not only improve data comparability across laboratories but also transparently reveal which approaches work only in high-competence model lines and which truly overcome recalcitrance, making them viable for integration into commercial wheat breeding programs.

## 9. Conclusions

Morphogenic factors emerge as central determinants of regeneration efficiency in wheat following genetic transformation. By reactivating conserved developmental and embryogenic programs, these regulators enable somatic cells to regain pluripotency and initiate organized plant development, thereby overcoming one of the major biological bottlenecks in wheat biotechnology. Controlled and transient manipulation of morphogenic genes such as *BBM*, *WUS/WUS2*, *GRF-GIF*, and *SERK* has been shown to significantly enhance embryogenic competence and increase transformation and genome-editing efficiency in plants. Importantly, precise temporal and spatial regulation of these factors is essential to avoid pleiotropic developmental defects while maximizing regenerative capacity. Overall, the strategic deployment of morphogenic regulators represents a powerful molecular approach to overcoming regeneration recalcitrance, facilitating genotype-independent transformation, and accelerating next-generation wheat improvement.

## Figures and Tables

**Table 1:** Signaling peptides and receptor-like kinases regulating meristem maintenance and embryogenic competence.

Signal/Family	Full Name/Family	Nature/Role	Main Biological Function	Effect on Tissue Competence	Typical Site of Action	References (APA)
CLE (CLV3)/CLV-WUS axis	*CLAVATA3/ESR-Related peptides*	Meristem-regulating peptides	Repress WUS to control the number of stem cells	Maintain stem cell population; balance between division and differentiation	Shoot apical meristem	[61,62,63]
PSK	*Phytosulfokine pentapeptides*	Sulfated growth peptides	Stimulate cell division and cell expansion	Enhance regenerative competence; support growth and renewal	Callus, growing tissues	[64,65,66]
RGF (GLV/CLEL)	*Root Meristem Growth Factor/GOLVEN peptides*	Root development-regulating peptides	Maintain root meristematic cell pool	Stabilize competence of root meristem; regulate meristem size	Root meristem	[67,68]
SERK	*Somatic Embryogenesis Receptor-Like Kinases*	LRR-RLK co-receptors	Support peptide/hormonal signaling	Crucial for embryogenic competence; mark competent cells	Embryogenic tissues, meristems	[69,70]
REF1	*REGENERATION FACTOR 1*	Wound-induced signaling peptide	Acts as a local wound signal promoting tissue and organ regeneration	Enhances regenerative competence; triggers callus formation and cellular reprogramming	Wounded tissues (leaf, stem)	[71]

**Table 2:** Pro-regenerative morphogenic regulators and their documented applications in wheat transformation and genome editing.

Morphogen/Gene	Function in Regeneration/Mechanism of Action	Application in Wheat + References
*GRF-GIF* (e.g., *TaGRF4-TaGIF1*)	Promotes cell proliferation, enhances regeneration competence, reduces dependence on exogenous cytokinin, and improves genome-editing efficiency	It was demonstrated that a GRF4-GIF1 chimeric protein substantially increases regeneration efficiency in wheat, allows for regeneration without external cytokinins, and supports CRISPR-Cas9 editing [38]. Transient expression of *TaGRF4-TaGIF1* complex increased regeneration and CRISPR-Cas genome-editing efficiency; the miR396-resistant version (*mTaGRF4-TaGIF1*) gave 2–9 × higher regeneration in 11 elite wheat cultivars [114].
*ZmBBM-ZmWUS2*	Morphogenic transcription factors that strongly promote somatic embryo formation and meristem initiation	The “QuickWheat” protocol using *ZmBBM* and *ZmWUS2* achieved high transformation efficiency (~58–75%) and reduced tissue culture time from ~80 days to ~50 days [115].
*TaWOX5*	*WUSCHEL-related homeobox* gene; enhances embryogenic competence and reduces genotype dependency	Overexpression of *TaWOX5* significantly increased transformation efficiency across multiple wheat genotypes [94].
*TaWOX14*	*WUSCHEL-related homeobox* gene; improves regeneration and genome-editing efficiency	*TaWOX14* overexpression enhanced regeneration and CRISPR-Cas9 editing efficiency in several wheat varieties [116].
*TaSERK* (*SERK* family)	Somatic Embryogenesis Receptor-Like Kinases; involved in embryogenic signaling and act as markers of embryogenic competence	*TaSERK1-3* are strongly expressed in embryogenic calli; their expression is induced by auxin or epibrassinolide, confirming their role in embryogenic competence [76].
*TaLAX1*	Auxin influx carrier/regulator of developmental reprogramming	Overexpression of *TaLAX1* strongly enhanced wheat regeneration and genetic transformation. The effect was linked to upregulation of *TaGRF* and *TaGIF1*, as well as cytokinin and auxin response genes [117].

## Data Availability

No new data were created or analyzed in this study. Data sharing is not applicable to this article.

## Supplementary Materials

The supporting information can be downloaded at https://www.mdpi.com/article/10.3390/ijms27031271/s1.

## References

[1] Ikeuchi M., Favero D.S., Sakamoto Y., Iwase A., Coleman D., Rymen B., Sugimoto K. (2019). Molecular Mechanisms of Plant Regeneration. Annu. Rev. Plant Biol..

[2] Li J., Zhang Q., Wang Z., Liu Q. (2024). The Roles of Epigenetic Regulators in Plant Regeneration: Exploring Patterns amidst Complex Conditions. Plant Physiol..

[3] Pasternak T.P., Steinmacher D. (2024). Plant Growth Regulation in Cell and Tissue Culture In Vitro. Plants.

[4] Sytar O., Fays M., Miseur J., Desoignies N., Gantait S., Chmielarz P. (2025). Advancements in In Vitro Seed Germination of Rare, Endangered Plants: Optimization Methods and Environmental Factors. Conservation of Plant Genetic Resources.

[5] Fan M., Xu C., Xu K., Hu Y. (2012). LATERAL ORGAN BOUNDARIES DOMAIN Transcription Factors Direct Callus Formation in Arabidopsis Regeneration. Cell Res..

[6] Meng W.J., Cheng Z.J., Sang Y.L., Zhang M.M., Rong X.F., Wang Z.W., Tang Y.Y., Zhang X.S. (2017). Type-B ARABIDOPSIS RESPONSE REGULATORs Specify the Shoot Stem Cell Niche by Dual Regulation of *WUSCHEL*. Plant Cell.

[7] Jeong Y.Y., Lee H.-Y., Kim S.W., Noh Y.-S., Seo P.J. (2021). Optimization of Protoplast Regeneration in the Model Plant Arabidopsis Thaliana. Plant Methods.

[8] Germanà M.A. (2011). Anther Culture for Haploid and Doubled Haploid Production. Plant Cell Tissue Organ Cult. PCTOC.

[9] Soriano M., Li H., Boutilier K. (2013). Microspore Embryogenesis: Establishment of Embryo Identity and Pattern in Culture. Plant Reprod..

[10] Ferrie A.M.R., Möllers C. (2011). Haploids and Doubled Haploids in Brassica Spp. for Genetic and Genomic Research. Plant Cell Tissue Organ Cult. PCTOC.

[11] Bhowmik P., Bilichak A. (2021). Advances in Gene Editing of Haploid Tissues in Crops. Genes.

[12] Impens L., Lorenzo C.D., Vandeputte W., Wytynck P., Debray K., Haeghebaert J., Herwegh D., Jacobs T.B., Ruttink T., Nelissen H. (2023). Combining Multiplex Gene Editing and Doubled Haploid Technology in Maize. New Phytol..

[13] Semagn K., Iqbal M., Alachiotis N., N’Diaye A., Pozniak C., Spaner D. (2021). Genetic Diversity and Selective Sweeps in Historical and Modern Canadian Spring Wheat Cultivars Using the 90K SNP Array. Sci. Rep..

[14] Hayta S., Smedley M.A., Demir S.U., Blundell R., Hinchliffe A., Atkinson N., Harwood W.A. (2019). An Efficient and Reproducible Agrobacterium-mediated Transformation Method for Hexaploid Wheat (*Triticum aestivum* L.). Plant Methods.

[15] Pellegrineschi A., Noguera L.M., Skovmand B., Brito R.M., Velazquez L., Salgado M.M., Hernandez R., Warburton M., Hoisington D. (2002). Identification of Highly Transformable Wheat Genotypes for Mass Production of Fertile Transgenic Plants. Genome.

[16] Zale J.M., Borchardt-Wier H., Kidwell K.K., Steber C.M. (2004). Callus Induction and Plant Regeneration from Mature Embryos of a Diverse Set of Wheat Genotypes. Plant Cell Tissue Organ Cult..

[17] Vasil V., Castillo A.M., Fromm M.E., Vasil I.K. (1992). Herbicide Resistant Fertile Transgenic Wheat Plants Obtained by Microprojectile Bombardment of Regenerable Embryogenic Callus. Bio/Technology.

[18] Cheng M., Fry J.E., Pang S., Zhou H., Hironaka C.M., Duncan D.R., Conner T.W., Wan Y. (1997). Genetic Transformation of Wheat Mediated by *Agrobacterium tumefaciens*. Plant Physiol..

[19] Sato K., Abe F., Mascher M., Haberer G., Gundlach H., Spannagl M., Shirasawa K., Isobe S. (2021). Chromosome-Scale Genome Assembly of the Transformation-Amenable Common Wheat Cultivar ‘Fielder’. DNA Res..

[20] Ishida Y., Tsunashima M., Hiei Y., Komari T., Wang K. (2015). Wheat (*Triticum aestivum* L.) Transformation Using Immature Embryos. Agrobacterium Protocols; Methods in Molecular Biology.

[21] Richardson T., Thistleton J., Higgins T.J., Howitt C., Ayliffe M. (2014). Efficient Agrobacterium Transformation of Elite Wheat Germplasm without Selection. Plant Cell Tissue Organ Cult..

[22] Ikeuchi M., Ogawa Y., Iwase A., Sugimoto K. (2016). Plant Regeneration: Cellular Origins and Molecular Mechanisms. Development.

[23] Kumar R., Mamrutha H.M., Kaur A., Venkatesh K., Grewal A., Kumar R., Tiwari V. (2017). Development of an Efficient and Reproducible Regeneration System in Wheat (*Triticum aestivum* L.). Physiol. Mol. Biol. Plants.

[24] Lopes M.S., El-Basyoni I., Baenziger P.S., Singh S., Royo C., Ozbek K., Aktas H., Ozer E., Ozdemir F., Manickavelu A. (2015). Exploiting Genetic Diversity from Landraces in Wheat Breeding for Adaptation to Climate Change. J. Exp. Bot..

[25] Amare A. (2025). In Vitro Callus Induction and Plant Regeneration in Bread Wheat (*Triticum aestivum* L.) Genotypes from Young Leave Base. J. Nat. Sci. Res..

[26] Wang X., Ren X., Yin G., Wang K., Li J., Du L., Xu H., Ye X. (2014). Effects of Environmental Temperature on the Regeneration Frequency of the Immature Embryos of Wheat (*Triticum aestivum* L.). J. Integr. Agric..

[27] Kavas M., Öktem H.A., Yücel M. (2008). Factors Affecting Plant Regeneration from Immature Inflorescence of Two Winter Wheat Cultivars. Biol. Plant..

[28] Dodig D., Nikolić R., Mitić N. (2008). Tissue culture response of different wheat genotypes, environmental effect and association with plant traits. Options Mediterr. Ser. A Mediterr. Semin..

[29] Gordon-Kamm B., Sardesai N., Arling M., Lowe K., Hoerster G., Betts S., Jones T. (2019). Using Morphogenic Genes to Improve Recovery and Regeneration of Transgenic Plants. Plants.

[30] Lowe K., Wu E., Wang N., Hoerster G., Hastings C., Cho M.-J., Scelonge C., Lenderts B., Chamberlin M., Cushatt J. (2016). Morphogenic Regulators *Baby boom* and *Wuschel* Improve Monocot Transformation. Plant Cell.

[31] Harrington S.A., Backhaus A.E., Fox S., Rogers C., Borrill P., Uauy C., Richardson A. (2020). A Heat-Shock Inducible System for Flexible Gene Expression in Cereals. Plant Methods.

[32] Vlad D., Abu-Jamous B., Wang P., Langdale J.A. (2019). A Modular Steroid-Inducible Gene Expression System for Use in Rice. BMC Plant Biol..

[33] Hiei Y., Ishida Y., Komari T. (2014). Progress of Cereal Transformation Technology Mediated by *Agrobacterium tumefaciens*. Front. Plant Sci..

[34] Bhalla P.L., Ottenhof H.H., Singh M.B. (2006). Wheat Transformation—An Update of Recent Progress. Euphytica.

[35] Vasil I.K. (2007). Molecular Genetic Improvement of Cereals: Transgenic Wheat (*Triticum aestivum* L.). Plant Cell Rep..

[36] Pérez-Piñeiro P., Gago J., Landín M., Gallego P.P., Ozden Ifti Y. (2012). Agrobacterium-mediated Transformation Of. Transgenic Plants—Advances and Limitations.

[37] Li F., Li X., Qiao M., Li B., Guo D., Zhang X., Min D. (2020). TaTCP-1, a Novel Regeneration-related Gene Involved in the Molecular Regulation of Somatic Embryogenesis in Wheat (*Triticum aestivum* L.). Front. Plant Sci..

[38] Debernardi J.M., Tricoli D.M., Ercoli M.F., Hayta S., Ronald P., Palatnik J.F., Dubcovsky J. (2020). A GRF–GIF Chimeric Protein Improves the Regeneration Efficiency of Transgenic Plants. Nat. Biotechnol..

[39] Nadolska-Orczyk A., Przetakiewicz A., Orczyk W. (2000). Transformacja genetyczna zbóż za pomocą Agrobacterium. Biotechnologia.

[40] Jones H.D., Doherty A., Wu H. (2005). Review of Methodologies and a Protocol for the Agrobacterium-Mediated Transformation of Wheat. Plant Methods.

[41] Bie X.M., Dong L., Li X.H., Wang H., Gao X.-Q., Li X.G. (2020). Trichostatin A and Sodium Butyrate Promotes Plant Regeneration in Common Wheat. Plant Signal. Behav..

[42] Zhao L., Yang Y., Chen J., Lin X., Zhang H., Wang H., Wang H., Bie X., Jiang J., Feng X. (2023). Dynamic Chromatin Regulatory Programs during Embryogenesis of Hexaploid Wheat. Genome Biol..

[43] Kulman K., Jobbágy K., Singh K., Szalai G., Pál M., Benczúr K., Pálmai T., Borbély P., Asghar M.A., Soltész A. (2025). Differences in the Improvement of Regeneration Ability of Wheat Calli by Ascorbate and H2O2 Through Modulation of Their Hormone and Metabolite Profile. J. Plant Growth Regul..

[44] Aydin M., Hossein Pour A., Haliloğlu K., Tosun M. (2016). Effect of Polyamines on Somatic Embryogenesis via Mature Embryo in Wheat. Turk. J. Biol..

[45] Bidabadi S.S., Jain S.M. (2020). Cellular, Molecular, and Physiological Aspects of In Vitro Plant Regeneration. Plants.

[46] Zimmerman J.L. (1993). Somatic Embryogenesis: A Model for Early Development in Higher Plants. Plant Cell.

[47] Morończyk J., Brąszewska A., Wójcikowska B., Chwiałkowska K., Nowak K., Wójcik A.M., Kwaśniewski M., Gaj M.D. (2022). Insights into the Histone Acetylation-mediated Regulation of the Transcription Factor Genes That Control the Embryogenic Transition in the Somatic Cells of Arabidopsis. Cells.

[48] Li J., Li Y., Ma L. (2021). Recent Advances in CRISPR/Cas9 and Applications for Wheat Functional Genomics and Breeding. aBIOTECH.

[49] Ye X., Shrawat A., Moeller L., Rode R., Rivlin A., Kelm D., Martinell B.J., Williams E.J., Paisley A., Duncan D.R. (2023). Agrobacterium-Mediated Direct Transformation of Wheat Mature Embryos through Organogenesis. Front. Plant Sci..

[50] Delporte F., Pretova A., Du Jardin P., Watillon B. (2014). Morpho-Histology and Genotype Dependence of in Vitro Morphogenesis in Mature Embryo Cultures of Wheat. Protoplasma.

[51] Skrzypek E., Szechyńska-Hebda M., Dąbrowska G., Goc A. (2008). The Role of Osmotic Stress During in Vitro Regeneration of *Triticum aestivum* L. and *Vicia faba* ssp. Minor. Zesz. Probl. Post. Nauk Rol..

[52] Ikeuchi M., Sugimoto K., Iwase A. (2013). Plant Callus: Mechanisms of Induction and Repression. Plant Cell.

[53] Raspor M., Motyka V., Kaleri A.R., Ninković S., Tubić L., Cingel A., Ćosić T. (2021). Integrating the Roles for Cytokinin and Auxin in De Novo Shoot Organogenesis: From Hormone Uptake to Signaling Outputs. Int. J. Mol. Sci..

[54] Nameth B., Dinka S.J., Chatfield S.P., Morris A., English J., Lewis D., Oro R., Raizada M.N. (2013). The Shoot Regeneration Capacity of Excised Arabidopsis Cotyledons Is Established during the Initial Hours after Injury and Is Modulated by a Complex Genetic Network of Light Signalling. Plant Cell Environ..

[55] Benderradji L., Brini F., Kellou K., Ykhlef N., Djekoun A., Masmoudi K., Bouzerzour H. (2012). Callus Induction, Proliferation, and Plantlets Regeneration of Two Bread Wheat (*Triticum aestivum* L.) Genotypes under Saline and Heat Stress Conditions. ISRN Agron..

[56] Iqbal M., Iqbal Raja N., Asif S., Ilyas N., Hussain M., Yasmeen F., Ejaz M., Asim Sultan M., Aslam S., Javed H. (2016). In Vitro Study of Callogenesis and Regeneration Potential of Elite Wheat (*Triticum aestivum* L.) Cultivars. Am. J. Plant Sci..

[57] Tamás C., Szűcs P., Rakszegi M., Tamás L., Bedő Z. (2004). Effect of Combined Changes in Culture Medium and Incubation Conditions on the Regeneration from Immature Embryos of Elite Varieties of Winter Wheat. Plant Cell Tissue Organ Cult..

[58] Han X., Deng Z., Liu H., Ji X. (2025). Current Advancement and Future Prospects in Simplified Transformation-Based Plant Genome Editing. Plants.

[59] Zhang S., Zhang R., Gao J., Song G., Li J., Li W., Qi Y., Li Y., Li G. (2021). CRISPR/Cas9-Mediated Genome Editing for Wheat Grain Quality Improvement. Plant Biotechnol. J..

[60] Wang K., Liu H., Du L., Ye X. (2017). Generation of Marker-free Transgenic Hexaploid Wheat via an *Agrobacterium*-mediated Co-transformation Strategy in Commercial Chinese Wheat Varieties. Plant Biotechnol. J..

[61] Brand U., Fletcher J.C., Hobe M., Meyerowitz E.M., Simon R. (2000). Dependence of Stem Cell Fate in *Arabidopsis* on a Feedback Loop Regulated by *CLV3* Activity. Science.

[62] Fletcher J.C. (2018). The CLV-WUS Stem Cell Signaling Pathway: A Roadmap to Crop Yield Optimization. Plants.

[63] Somssich M., Je B.I., Simon R., Jackson D. (2016). CLAVATA-WUSCHEL Signaling in the Shoot Meristem. Development.

[64] Zhang P., Guo L., Long J., Chen T., Gao W., Zhang X., Ma J., Wang P., Yang D. (2024). Genome-Wide Identification of Phytosulfokine (PSK) Peptide Family Reveals TaPSK1 Gene Involved in Grain Development of Wheat (*Triticum aestivum* L.). Chem. Biol. Technol. Agric..

[65] Kaufmann C., Motzkus M., Sauter M. (2017). Phosphorylation of the Phytosulfokine Peptide Receptor PSKR1 Controls Receptor Activity. J. Exp. Bot..

[66] Hao Z., Wu H., Zheng R., Li R., Zhu Z., Chen Y., Lu Y., Cheng T., Shi J., Chen J. (2023). The Plant Peptide Hormone Phytosulfokine Promotes Somatic Embryogenesis by Maintaining Redox Homeostasis in *Cunninghamia lanceolata*. Plant J..

[67] Fedoreyeva L.I. (2023). Molecular Mechanisms of Regulation of Root Development by Plant Peptides. Plants.

[68] Lu S., Xiao F. (2024). Small Peptides: Orchestrators of Plant Growth and Developmental Processes. Int. J. Mol. Sci..

[69] Hecht V., Vielle-Calzada J.-P., Hartog M.V., Schmidt E.D.L., Boutilier K., Grossniklaus U., De Vries S.C. (2001). The Arabidopsis Somatic Embryogenesis Receptor Kinase 1 Gene Is Expressed in Developing Ovules and Embryos and Enhances Embryogenic Competence in Culture. Plant Physiol..

[70] Karim R., Tan Y.S., Singh P., Khalid N., Harikrishna J.A. (2018). Expression and DNA Methylation of SERK, BBM, LEC2 and WUS Genes in in Vitro Cultures of *Boesenbergia rotunda* (L.) Mansf. Physiol. Mol. Biol. Plants.

[71] Yang W., Zhai H., Wu F., Deng L., Chao Y., Meng X., Chen Q., Liu C., Bie X., Sun C. (2024). Peptide REF1 Is a Local Wound Signal Promoting Plant Regeneration. Cell.

[72] Li Z., Liu D., Xia Y., Li Z., Niu N., Ma S., Wang J., Song Y., Zhang G. (2019). Identification and Functional Analysis of the CLAVATA3/EMBRYO SURROUNDING REGION (CLE) Gene Family in Wheat. Int. J. Mol. Sci..

[73] Yang W., Feng M., Yu K., Cao J., Cui G., Zhang Y., Peng H., Yao Y., Hu Z., Ni Z. (2025). The TaCLE24b Peptide Signaling Cascade Modulates Lateral Root Development and Drought Tolerance in Wheat. Nat. Commun..

[74] Matsubayashi Y., Hanai H., Hara O., Sakagami Y. (1996). Active Fragments and Analogs of the Plant Growth Factor, Phytosulfokine: Structure–Activity Relationships. Biochem. Biophys. Res. Commun..

[75] Albrecht C., Russinova E., Kemmerling B., Kwaaitaal M., De Vries S.C. (2008). Arabidopsis SOMATIC EMBRYOGENESIS RECEPTOR KINASE Proteins Serve Brassinosteroid-Dependent and -Independent Signaling Pathways. Plant Physiol..

[76] Singh A., Khurana P. (2017). Ectopic Expression of *Triticum aestivum* SERK Genes (TaSERKs) Control Plant Growth and Development in Arabidopsis. Sci. Rep..

[77] Singla B., Khurana J.P., Khurana P. (2008). Characterization of Three Somatic Embryogenesis Receptor Kinase Genes from Wheat, *Triticum aestivum*. Plant Cell Rep..

[78] Nolan K.E., Kurdyukov S., Rose R.J. (2009). Expression of the SOMATIC EMBRYOGENESIS RECEPTOR-LIKE KINASE1 (SERK1) Gene Is Associated with Developmental Change in the Life Cycle of the Model Legume *Medicago truncatula*. J. Exp. Bot..

[79] Boutilier K., Offringa R., Sharma V.K., Kieft H., Ouellet T., Zhang L., Hattori J., Liu C.-M., Van Lammeren A.A.M., Miki B.L.A. (2002). Ectopic Expression of BABY BOOM Triggers a Conversion from Vegetative to Embryonic Growth. Plant Cell.

[80] Mookkan M., Nelson-Vasilchik K., Hague J., Zhang Z.J., Kausch A.P. (2017). Selectable Marker Independent Transformation of Recalcitrant Maize Inbred B73 and Sorghum P898012 Mediated by Morphogenic Regulators BABY BOOM and WUSCHEL2. Plant Cell Rep..

[81] Horstman A., Bemer M., Boutilier K. (2017). A Transcriptional View on Somatic Embryogenesis. Regeneration.

[82] Omidbakhshfard M.A., Proost S., Fujikura U., Mueller-Roeber B. (2015). Growth-Regulating Factors (GRFs): A Small Transcription Factor Family with Important Functions in Plant Biology. Mol. Plant.

[83] Yang Z., Zhao M., Zhang X., Gu L., Li J., Ming F., Wang M., Wang Z. (2024). MIR396-GRF/GIF Enhances in Planta Shoot Regeneration of *Dendrobium catenatum*. BMC Genom..

[84] Bull T., Debernardi J., Reeves M., Hill T., Bertier L., Van Deynze A., Michelmore R. (2023). GRF–GIF Chimeric Proteins Enhance in Vitro Regeneration and Agrobacterium-Mediated Transformation Efficiencies of Lettuce (*Lactuca* spp.). Plant Cell Rep..

[85] Komatsu M., Maekawa M., Shimamoto K., Kyozuka J. (2001). The LAX1 and FRIZZY PANICLE 2 Genes Determine the Inflorescence Architecture of Rice by Controlling Rachis-Branch and Spikelet Development. Dev. Biol..

[86] Oikawa T., Kyozuka J. (2009). Two-Step Regulation of *LAX PANICLE1* Protein Accumulation in Axillary Meristem Formation in Rice. Plant Cell.

[87] Wang Y., Zhang T., Wang R., Zhao Y. (2018). Recent Advances in Auxin Research in Rice and Their Implications for Crop Improvement. J. Exp. Bot..

[88] Ikeuchi M., Rymen B., Sugimoto K. (2020). How Do Plants Transduce Wound Signals to Induce Tissue Repair and Organ Regeneration?. Curr. Opin. Plant Biol..

[89] Iwase A., Mitsuda N., Koyama T., Hiratsu K., Kojima M., Arai T., Inoue Y., Seki M., Sakakibara H., Sugimoto K. (2011). The AP2/ERF Transcription Factor WIND1 Controls Cell Dedifferentiation in Arabidopsis. Curr. Biol..

[90] Iwase A., Harashima H., Ikeuchi M., Rymen B., Ohnuma M., Komaki S., Morohashi K., Kurata T., Nakata M., Ohme-Takagi M. (2017). WIND1 Promotes Shoot Regeneration through Transcriptional Activation of *ENHANCER OF SHOOT REGENERATION1* in Arabidopsis. Plant Cell.

[91] Iwase A., Kondo Y., Laohavisit A., Takebayashi A., Ikeuchi M., Matsuoka K., Asahina M., Mitsuda N., Shirasu K., Fukuda H. (2021). WIND Transcription Factors Orchestrate Wound-induced Callus Formation, Vascular Reconnection and Defense Response in Arabidopsis. New Phytol..

[92] Canher B., Heyman J., Savina M., Devendran A., Eekhout T., Vercauteren I., Prinsen E., Matosevich R., Xu J., Mironova V. (2020). Rocks in the Auxin Stream: Wound-induced Auxin Accumulation and *ERF115* Expression Synergistically Drive Stem Cell Regeneration. Proc. Natl. Acad. Sci. USA.

[93] Sarkar A.K., Luijten M., Miyashima S., Lenhard M., Hashimoto T., Nakajima K., Scheres B., Heidstra R., Laux T. (2007). Conserved Factors Regulate Signalling in Arabidopsis Thaliana Shoot and Root Stem Cell Organizers. Nature.

[94] Wang K., Shi L., Liang X., Zhao P., Wang W., Liu J., Chang Y., Hiei Y., Yanagihara C., Du L. (2022). The Gene TaWOX5 Overcomes Genotype Dependency in Wheat Genetic Transformation. Nat. Plants.

[95] Xu P., Zhong Y., Xu A., Liu B., Zhang Y., Zhao A., Yang X., Ming M., Cao F., Fu F. (2024). Application of Developmental Regulators for Enhancing Plant Regeneration and Genetic Transformation. Plants.

[96] Drisch R.C., Stahl Y. (2015). Function and Regulation of Transcription Factors Involved in Root Apical Meristem and Stem Cell Maintenance. Front. Plant Sci..

[97] Lee K., Wang K. (2023). Strategies for Genotype-Flexible Plant Transformation. Curr. Opin. Biotechnol..

[98] Tian H., Wabnik K., Niu T., Li H., Yu Q., Pollmann S., Vanneste S., Govaerts W., Rolčík J., Geisler M. (2014). WOX5–IAA17 Feedback Circuit-Mediated Cellular Auxin Response Is Crucial for the Patterning of Root Stem Cell Niches in Arabidopsis. Mol. Plant.

[99] Si X., Wang W., Wang K., Liu Y., Bai J., Meng Y., Zhang X., Liu H. (2021). A Sheathed Spike Gene, TaWUS-like Inhibits Stem Elongation in Common Wheat by Regulating Hormone Levels. Int. J. Mol. Sci..

[100] Lowe K., La Rota M., Hoerster G., Hastings C., Wang N., Chamberlin M., Wu E., Jones T., Gordon-Kamm W. (2018). Rapid Genotype “Independent” *Zea mays* L. (Maize) Transformation via Direct Somatic Embryogenesis. In Vitro Cell. Dev. Biol.-Plant.

[101] Jiang Y., Liu S., An X. (2024). Functional Mechanisms and the Application of Developmental Regulators for Improving Genetic Transformation in Plants. Plants.

[102] Youngstrom C., Wang K., Lee K. (2025). Unlocking Regeneration Potential: Harnessing Morphogenic Regulators and Small Peptides for Enhanced Plant Engineering. Plant J..

[103] Hoerster G., Wang N., Ryan L., Wu E., Anand A., McBride K., Lowe K., Jones T., Gordon-Kamm B. (2020). Use of Non-Integrating Zm-Wus2 Vectors to Enhance Maize Transformation: Non-Integrating WUS2 Enhances Transformation. In Vitro Cell. Dev. Biol.-Plant.

[104] Gaj M.D., Zhang S., Harada J.J., Lemaux P.G. (2005). Leafy Cotyledon Genes Are Essential for Induction of Somatic Embryogenesis of Arabidopsis. Planta.

[105] Nowak K., Wójcik A.M., Konopka K., Jarosz A., Dombert K., Gaj M.D. (2024). miR156-SPL and miR169-NF-YA Modules Regulate the Induction of Somatic Embryogenesis in Arabidopsis via LEC- and Auxin-Related Pathways. Int. J. Mol. Sci..

[106] Martínez M., Corredoira E. (2024). Recent Advances in Plant Somatic Embryogenesis: Where We Stand and Where to Go?. Int. J. Mol. Sci..

[107] Brand A., Quimbaya M., Tohme J., Chavarriaga-Aguirre P. (2019). Arabidopsis LEC1 and LEC2 Orthologous Genes Are Key Regulators of Somatic Embryogenesis in Cassava. Front. Plant Sci..

[108] Wójcikowska B., Jaskóła K., Gąsiorek P., Meus M., Nowak K., Gaj M.D. (2013). LEAFY COTYLEDON2 (LEC2) Promotes Embryogenic Induction in Somatic Tissues of Arabidopsis, via YUCCA-Mediated Auxin Biosynthesis. Planta.

[109] Braybrook S., Harada J. (2008). LECs Go Crazy in Embryo Development. Trends Plant Sci..

[110] Aida M., Beis D., Heidstra R., Willemsen V., Blilou I., Galinha C., Nussaume L., Noh Y.-S., Amasino R., Scheres B. (2004). The PLETHORA Genes Mediate Patterning of the Arabidopsis Root Stem Cell Niche. Cell.

[111] Galinha C., Hofhuis H., Luijten M., Willemsen V., Blilou I., Heidstra R., Scheres B. (2007). PLETHORA Proteins as Dose-Dependent Master Regulators of Arabidopsis Root Development. Nature.

[112] Duan H., Maren N.A., Ranney T.G., Liu W. (2022). New Opportunities for Using *WUS*/*BBM* and *GRF-GIF* Genes to Enhance Genetic Transformation of Ornamental Plants. Ornam. Plant Res..

[113] Kadri A., Grenier De March G., Guerineau F., Cosson V., Ratet P. (2021). WUSCHEL Overexpression Promotes Callogenesis and Somatic Embryogenesis in *Medicago truncatula* Gaertn. Plants.

[114] Qiu F., Xing S., Xue C., Liu J., Chen K., Chai T., Gao C. (2022). Transient Expression of a TaGRF4-TaGIF1 Complex Stimulates Wheat Regeneration and Improves Genome Editing. Sci. China Life Sci..

[115] Johnson K., Chu U.C., Anthony G., Wu E., Che P., Jones T.J. (2023). Rapid and Highly Efficient Morphogenic Gene-Mediated Hexaploid Wheat Transformation. Front. Plant Sci..

[116] Wang D., Guo Y., Liu M., Liu H. (2025). The Function of TaWOX14 in Wheat Genetic Transformation. Plant Cell Rep..

[117] Yu Y., Yu H., Peng J., Yao W.J., Wang Y.P., Zhang F.L., Wang S.R., Zhao Y., Zhao X.Y., Zhang X.S. (2024). Enhancing Wheat Regeneration and Genetic Transformation through Overexpression of TaLAX1. Plant Commun..

[118] Aesaert S., Impens L., Coussens G., Van Lerberge E., Vanderhaeghen R., Desmet L., Vanhevel Y., Bossuyt S., Wambua A.N., Van Lijsebettens M. (2022). Optimized Transformation and Gene Editing of the B104 Public Maize Inbred by Improved Tissue Culture and Use of Morphogenic Regulators. Front. Plant Sci..

[119] Che P., Wu E., Simon M.K., Anand A., Lowe K., Gao H., Sigmund A.L., Yang M., Albertsen M.C., Gordon-Kamm W. (2022). Wuschel2 Enables Highly Efficient CRISPR/Cas-Targeted Genome Editing during Rapid de Novo Shoot Regeneration in Sorghum. Commun. Biol..

