# Supplementary material for: Molecular Regulators of In Vitro Regeneration in Wheat: Roles of Morphogenic Factors in Transformation, Genome Editing, and Breeding
Source: Int J Mol Sci. 2026 Jan 27;27(3):1271. doi: 10.3390/ijms27031271 (PMC12898219; doi:10.3390/ijms27031271)
Supplement: Supplementary file 1 [file ijms-27-01271-s001.zip › ijms-4061100-supplementary.pdf]

Supplementary Material

Supplementary Figures for IJMS manuscript

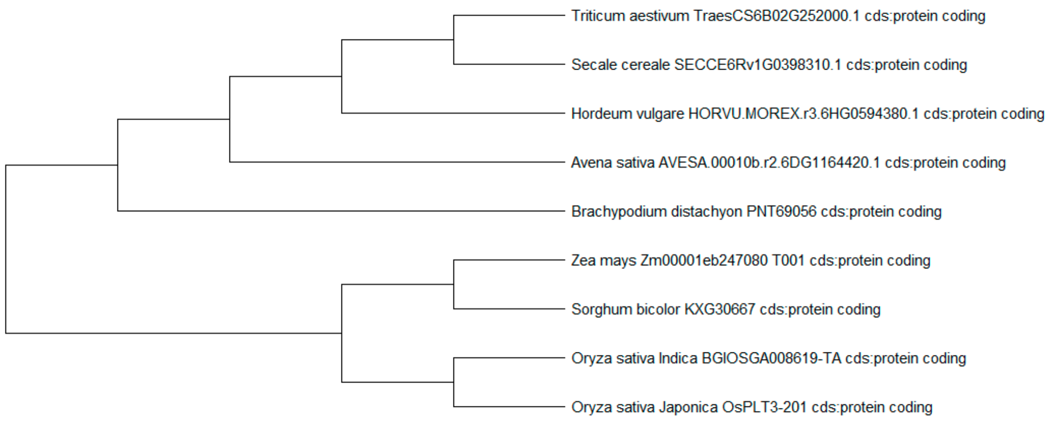

Figure S1 Phylogenetic relationships among BBM orthologs from major cereal species

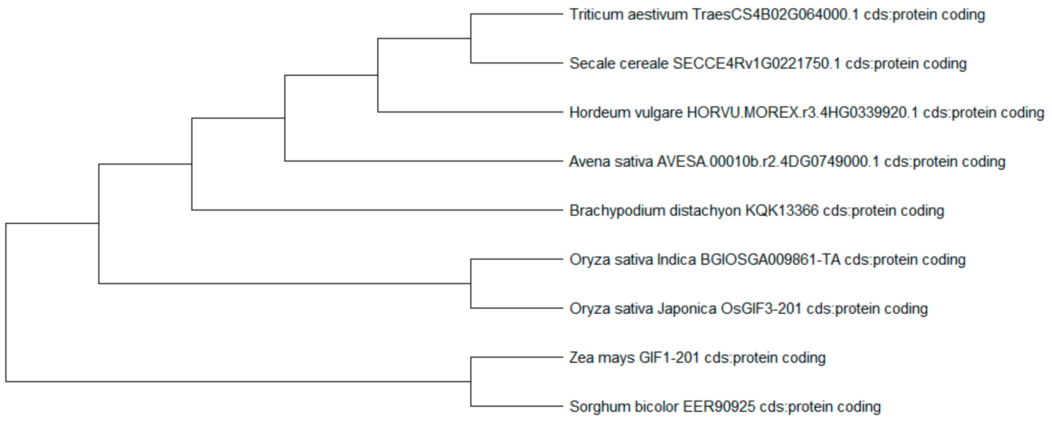

Figure S2 Phylogenetic relationships among GIF orthologs from major cereal species

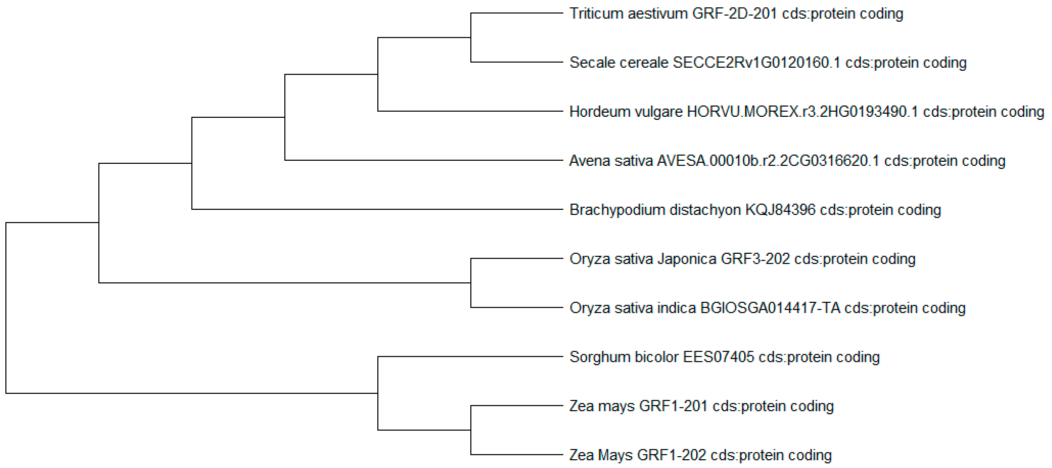

Figure S3 Phylogenetic relationships among GRF orthologs from major cereal species

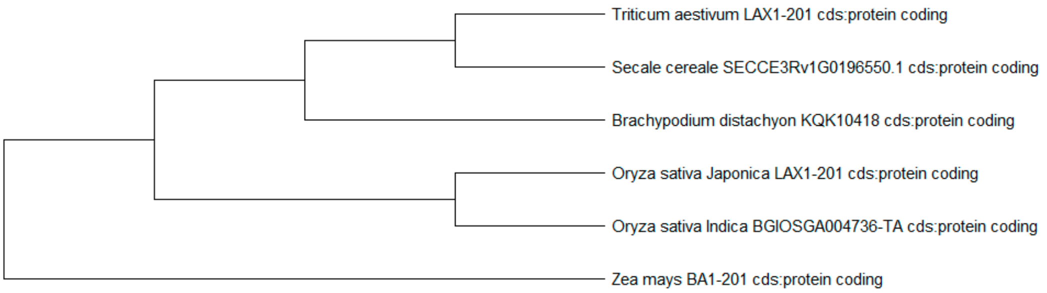

Figure S4 Phylogenetic relationships among LAX1 orthologs from major cereal species

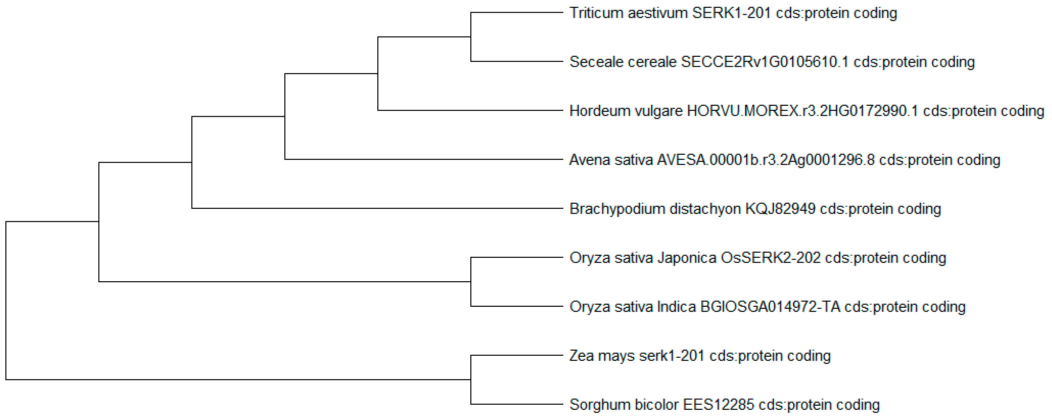

Figure S5 Phylogenetic relationships among SERK orthologs from major cereal species

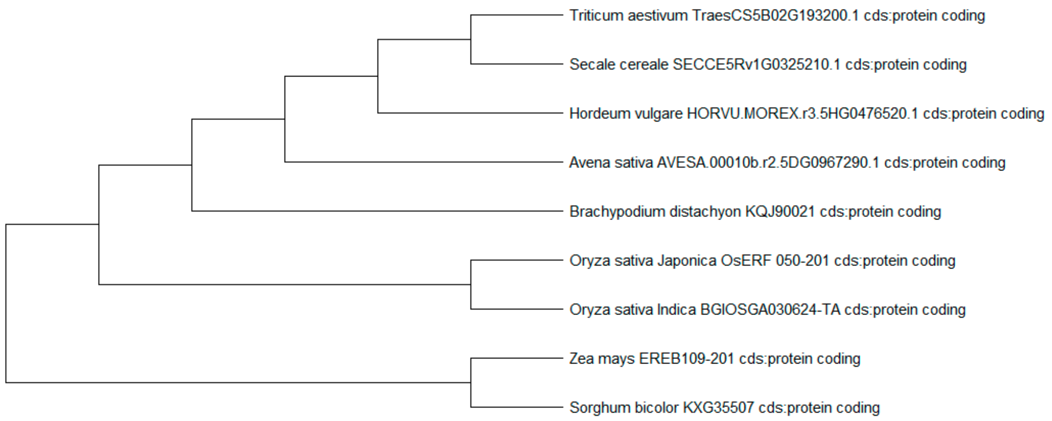

Figure S6 Phylogenetic relationships among WIND1 orthologs from major cereal species

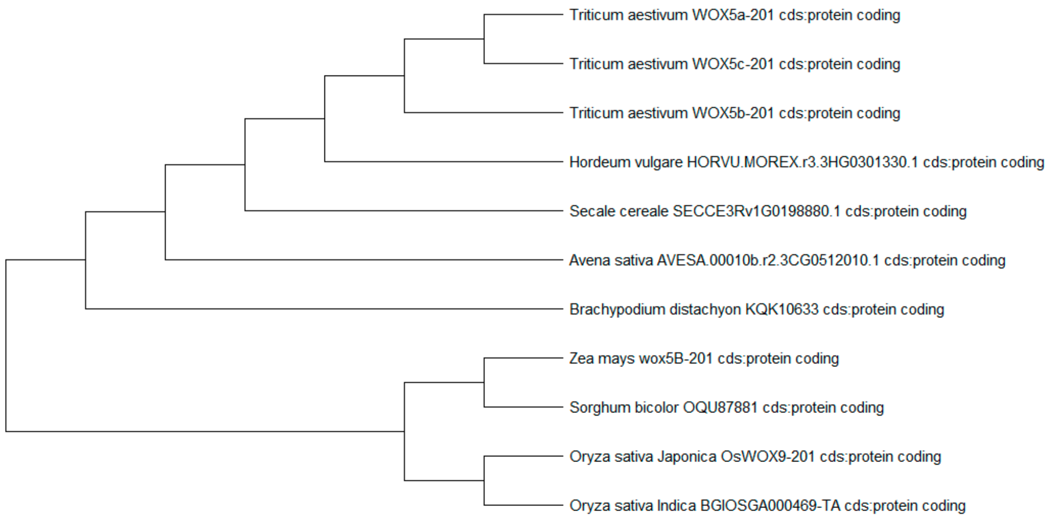

Figure S7 Phylogenetic relationships among WOX5 orthologs from major cereal species

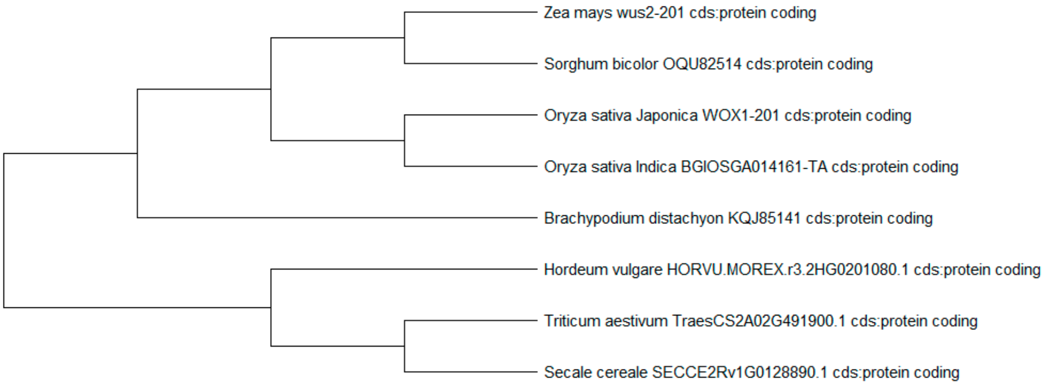

Figure S8 Phylogenetic relationships among WUS2 orthologs from major cereal species

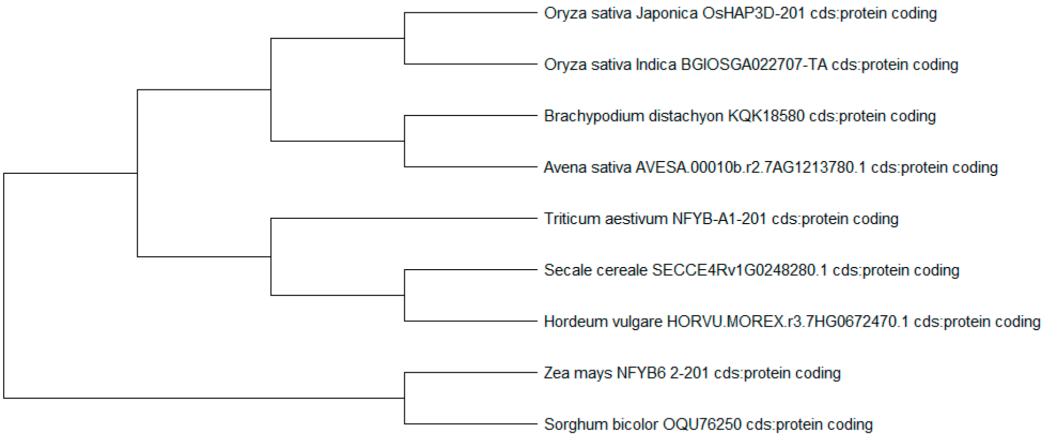

Figure S9 Phylogenetic relationships among LEC1 orthologs from major cereal species

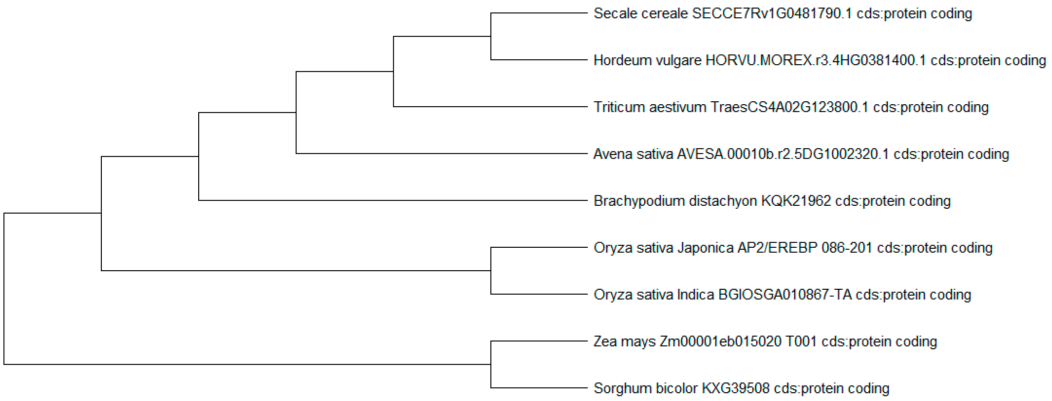

Figure S10 Phylogenetic relationships among PLT/AIL orthologs from major cereal species
